# Sex-specific analysis of traumatic brain injury events: applying computational and data visualization techniques to inform prevention and management

**DOI:** 10.1186/s12874-021-01493-6

**Published:** 2022-01-30

**Authors:** Tatyana Mollayeva, Andrew Tran, Vincy Chan, Angela Colantonio, Michael D. Escobar

**Affiliations:** 1 KITE-Toronto Rehabilitation Institute, University Health Network, Toronto, Ontario Canada; 2 Department of Occupational Science & Occupational Therapy, University of Toronto, Toronto, Ontario Canada; 3 Rehabilitation Sciences Institute, Temerty Faculty of Medicine, University of Toronto, Toronto, Ontario Canada; 4 Acquired Brain Injury Research Lab, University of Toronto, Toronto, Ontario Canada; 5 Dalla Lana School of Public Health, University of Toronto, Toronto, Canada Ontario; 6 Trinity College Institute of Neuroscience, Global Brain Health Institute, Dublin, Ireland; 7 Institute of Health Policy, Management and Evaluation, University of Toronto, Toronto, Ontario Canada

**Keywords:** Data-driven research, Environment, Health analytics, Human factors, Physical force agent, The Haddon matrix, Sex

## Abstract

**Background:**

The interplay of host, agent, and environment implicated in traumatic brain injury (TBI) events is difficult to account for in hypothesis-driven research. Data-driven analysis of injury data can enable insight into injury events in novel ways. This research dissected complex and multidimensional data at the time of the TBI event by exploiting data mining and information visualization methods.

**Methods:**

We drew upon population-based decade-long health administrative data collected through the routine operation of the publicly funded health system in Ontario, Canada. We applied a computational approach to categorize health records of 235,003 patients with TBI versus the same number of reference patients without TBI, individually matched based on sex, age, place of residence, and neighbourhood income quantile. We adopted the basic concepts of the Haddon Matrix (host, agent, environment) to organize emerging factors significantly related to TBI versus non-TBI events. To explore sex differences, the data of male and female patients with TBI were plotted on heatmaps and clustered using hierarchical clustering algorithms.

**Results:**

Based on detected similarities, the computational technique yielded 34 factors on which individual TBI-event codes were loaded, allowing observation of a set of definable patterns within the host, the agent, and the environment. Differences in the patterns of host, agent and environment were found between male and female patients with TBI, which are currently not identified based on data from injury surveillance databases. The results were internally validated.

**Conclusions:**

The study outlines novel areas for research relevant to TBI and offers insight into how computational and visual techniques can be applied to advance the understanding of TBI event. Results highlight unique aspects of sex differences of the host and agent at the injury event, as well as differences in exposure to adverse social and environmental circumstances, which can be a function of gender, aiding in future studies of injury prevention and gender-transformative care.

**Supplementary Information:**

The online version contains supplementary material available at 10.1186/s12874-021-01493-6.

## Background

Traumatic brain injury (TBI), defined as an alteration in brain function or other evidence of brain pathology resulting from an external force, is a significant public health problem globally [1, 2]. It is estimated that 50–60 million people worldwide are affected by TBI annually, and it is predicted that close to 50% of the global population will sustain a TBI in their lifetime, costing the international economy approximately US$400 billion each year [1, 2]. Given an estimated standardized gross world product of US $73.7 trillion, the economic cost of TBI represents 0.5% of the annual global output [1, 3].

A majority of the TBIs sustained are mild in severity (mild TBI), occurring at an annual incidence of 224 (95% CI: 120–418) per 100,000 person-years, almost ten times higher than the incidence of moderate TBI and 17 times higher than that of severe TBI [1, 4]. Historically, TBI was viewed as predominantly a male injury sustained in war conflicts, occupational accidents, and sports; as a gender-based division of labour and leisure activities dissipate and with more attention on concussion, the prevalence of TBI in female persons increases, matching that observed in males in later life [5]. Considering the increasing burden of TBI in both male and female persons [4, 5], the World Health Organization and the federal agencies of many developed countries have identified TBI prevention as a priority, while also setting requirements for the inclusion of biological sex and sociocultural process of gender into research hypotheses [5–7]. As a result, the field of TBI has witnessed tremendous transformation and growth in both scope and depth over the past decade [5]. Among the most profound changes that have come from the research is the growing acceptance of TBI as an event that is most likely not random and is unequally distributed in the population, based on health status and risk factors preceding the injury [5, 8]. For example, the presence of substance-use disorders, epilepsy, and medication effects can potentiate or modify the risks associated with adverse behaviors (e.g., assault and domestic abuse) or falls [3, 7–9], which may affect male and female patients differently and have different implications for an injury event with a TBI outcome. Adding to these risks, many known adverse social determinants of health (e.g., socioeconomic disparity, racial and gender inequality) have been implicated in vulnerability to injury and decreased help-seeking after injury due to depleted psychosocial reserves [10–13]. A recent study highlighted that disorders of the elderly and medical issues, mental health disorders, and metabolic disorders preceding a TBI event were more frequently occurring in female patients than males; these disorders are implicated in more frequent falls due to increased frailty, especially in older persons [8]. In male patients, relatively higher occurrence of injuries from sharp instruments and moving machinery and emergencies and adversities due to substance use within five years preceding TBI [8] highlight the links to a gender-based division of labour and high risk behaviours, which might be reflected in injuries as a result of being struck by or against an object and interpersonal violence; all of which have implications for clinical and prevention contexts, and TBI surveillance system overall [4, 5].

The interplay of many clinical, social, and environmental factors implicated in injury events is difficult to account for in hypothesis-driven research. For a long time, scientists have been debating the role of hypothesis testing in injury surveillance. Such concerns reflect the centrality of hypothesis-driven research around humans’ level of expertise and knowledge, and the linear process in testing the hypothesis-driven associations. Many would argue that focusing primarily on a hypothesis-driven account diminishes the scientific curiosity and also stops scientific progress by encouraging scientists to focus on narrowly defined questions that can be posed as testable hypotheses. This issue is particularly relevant in complex injuries such as TBI, where the hypothesis-driven approach might stop new directions of analysis (e.g., the understanding the interrelatedness between personal, injury-related and environmental factors in injury or the nature-nurture interaction) and preclude identification of important relationships among many interacting variables in a non-linear fashion. Most recently, data-driven approaches to the analysis of injury data have emerged to enable understanding of TBI events in novel ways, providing evidence that TBI is caused by the interplay of three categories of factors: the host (i.e., variables characterizing the persons involved in injury with TBI outcome), the agent (i.e., an injury-producing agent that reaches the host and allows the transfer of energy in injurious amounts to cause TBI), and the environment (i.e., the context, circumstance, and conditions that may influence, directly or indirectly, the occurrence of injury, consisted of physical and social, including emergency medical services systems) [14–20], known as the epidemiologic triad in the Haddon Matrix [21–23]. The Haddon Matrix consists of three columns (host, agent, and environment) and three rows (pre-event, event, post-event phases) [21, 23]. This study focused on the event phase.

To date, most of data-driven studies that focused on the TBI event phase characterized the person (the host) who sustained the injury or how the injury-producing agent/its energy reached the host in injurious amounts (the agent of injury) as determinants of TBI outcomes (Supplementary Table 1). For example, Hernandes Rocha and colleagues (2017) studied the hospital registry data of 3138 patients utilizing a machine learning technique and reported that higher Glasgow Coma Scale (GCS) score, pulse oximetry values, and having a surgery were predictive of good recovery, while advanced patient age and injuries sustained in a motor vehicle collision (MVC) were predictive of poor recovery [16]. Recently, Folweiler and colleagues (2020) proposed a hybrid machine learning technique to identify phenotypes of patients with TBI during the acute injury phase and reported that when patients were grouped by baseline GCS score, no differences were observed in their clinical profiles or outcomes [17], suggesting that the GCS score may not be fool-proof for all TBI severities and that other factors within the human ecosystem, the agent and the environment [24], are essential to consider in TBI prevention strategies [17]. This notion is further supported by recent population-based studies which have revealed that multiple chronic disorders, alcohol and prescribed drug toxicity, and exposures to environmental adversities preceding a TBI event have implications for TBI outcomes and cost of publicly funded healthcare, affecting male and female patients differently [8, 25–27]. These differences motivated the present study’s design, which aimed to utilize the key concepts of host-agent-environment of the Haddon Matrix [21, 22] to analyze the data of patients with a diagnosis of TBI through a sex lens in the event phase.

Here, we utilized computational approaches to sequence host, agent, and environment data at the injury event from International Statistical Classification of Diseases and Related Health Problems, Tenth Revision Canadian Enhancement (ICD-10-CA) codes [28]. Given recent reports concerning sex and gender differences in TBI [8], we anticipated that data captured during the injury event will differ between the sexes, rooted in biological and social differences between male and female patients [30–32, Supplementary Table 2].

## Methods

The research ethics boards approved the study protocol at the clinical (University Health Network) and academic institutions (University of Toronto) with which the authors are affiliated. We followed The Strengthening the Reporting of Observational Studies in Epidemiology (STROBE) and Sex and Gender Equity in Research (SAGER) guidelines to conduct and report our research.

### Data

Data utilized in this study were acquired from ICES [29], which collects and stores health administrative data on publicly funded services provided to residents of Ontario, Canada, including information on acute care hospitalizations and emergency department (ED) visits. Ontario is Canada’s most populous province, comprising approximately 40% of Canada’s population [30]. Universal healthcare in Ontario covers all medically necessary healthcare services at the point of care [31]. The data were linked deterministically at the individual patient level through a unique, encoded identifier to other administrative data based on name, sex, date of birth, and postal code. Information in hospital and ED databases is recorded using the Canadian coding standards for the ICD-10-CA [28]. Each ICD-10-CA code consists of a combination of alphanumeric characters that characterize broad diagnosis categories arranged hierarchically, with the code length ranging from three to six characters. The first three characters designate the diagnosis category, which is the same as the World Health Organization’s ICD-10 international standard for reporting diseases and health conditions [28].

### Materials

We used a previously established cohort of patients discharged from acute care (identified in the Discharge Abstract Database, DAD) and from the ED (identified in the National Ambulatory Care Reporting System, NACRS) between fiscal years 2007/2008 and 2015/2016 with a diagnostic code for TBI (ICD-10-CA codes S02.0, S02.1, S02.3, S02.7, S02.8, S02.9, S04.0, S07.1, and S06) [8, 26, 27]; these patients comprised the TBI cohort in the present study. Data on patient demographics and main and secondary diagnoses, conditions, problems, and injury circumstances were collected for each patient. We followed previously published severity classifications to assign TBI injury severity to every patient [32, 33]. External causes of injury were established using Centers for Disease Control and Prevention’s (CDC; United States) injury matrix, divided into falls, struck by/against an object, MVC, and assaults [34]. We additionally identified sports-related injuries based on the Association of Public Health Epidemiologists in Ontario (APHEO) criteria [35]. We selected a 10% random sample of patients discharged from acute care hospitals or the ED during the same study period for any reason other than TBI, individually matched to patients with TBI on sex, age, place of residence (urban vs. rural), and income quintile; these patients comprised the reference patients’ cohort.

### Statistical analyses

To define the TBI event window, a histogram for the days from the index date was constructed. The index date for patients with TBI was their first TBI-related hospital and/or ED visit during the study period (fiscal years between 2007/2008 and 2015/2016). A peak was observed around the index date with the frequency dropping to a stationary point 30 days preceding, and following, the index date. This 61-day window, therefore, was determined as a TBI event phase and was the focus of this study. A similar event-phase window was defined for patients in the reference cohort, with the exception that the midpoint of each patient’s hospital visits was selected as an index date.

To analyze the health status of the host, agent, and the environment at the TBI event phase and gain insight into their observed relationships, we analyzed all ICD-10-CA codes recorded across the 10 and 25 diagnosis fields of the NACRS and the DAD respectively, at the first three-character level, for patients with TBI and their matched reference patients. We converted all ICD-10-CA codes (i.e., 2600) into binary variables, except for provisional codes for research and temporary assignments, U98 and U99. These 2600 binary ICD-10-CA code variables were tested for significant associations with TBI diagnosis codes using a matched McNemar test with correction for multiple testing [36]. The Benjamini-Yekutieli (BY) method was applied to acquire a set of codes controlled at a False Discovery Rate (FDR) of 5% [37]. For a set of ordered observed *p*-values, the BY method rejects *k* null hypotheses ${H}_{(1)}^0\cdots {H}_{(k)}^0$ where *k* is defined as

1 $$k=\max \left\{i:{p}_{(i)}\le \frac{i}{m{\sum}_{i=1}^mi}q\right\}$$

Where *i* is the *p*-value ranking, *m* is the total number of tests conducted, and *q* is the level of significance (set at 0.05 or 5% for this study).

Any of the three-alphanumeric digit ICD-10-CA codes that fulfilled the following criteria were retained for further analysis: a) identified as significant using McNemar tests following BY correction, and b) had a calculated odds ratio (OR) greater than one, compared with the reference cohort. The procedure was first performed using the training dataset and then repeated using the validation dataset. Only codes that were significant in both the training and validation sets were retained for further factor analysis using the principal components method [8, 38]. The optimal number of factors was determined by the breakpoint on the scree plot, eigenvalue, the highest cumulative proportion of variance accounted for, and via a conditional logistic regression looped across all factors covering the greatest area under the receiver operating characteristic curves [39]. Word clouds were generated to visualize the sex-specific frequencies, proportion, and ORs of the factors, where the size of the words indicated the magnitudes of these values [40].

Each of the factors at the injury event was defined as host, agent, or environment-related by studying its comprising ICD codes (See Supplementary Table 3). The designation of the host was given to factors whose ICD-10-CA codes characterized health status of the male and female patients at the injury event and which may have influenced a person’s susceptibility to an injury event or care at the time of injury. These factors consisted of ICD codes of chronic conditions and risk-taking, alcohol, and drug use captured at the injury event as additional diagnoses to TBI diagnosis. The designation of agent was given to factors composed of ICD-10-CA codes that described how the injury-producing agent reached the host, allowing the transfer of physical energy in such injurious amounts to result in a TBI. The Environment designation comprised all the codes constituting the context, circumstance, and conditions that have influenced, directly or indirectly, the occurrence of injury and health care at the injury phase [41, 42]. Situations where codes forming the factor comprised of a combination of codes describing host and agent, or host and environment, were designated as host/environment (H/E) and host/agent (H/A), respectively [41, 42].

The conditional logistic regression models were then built using designated binary factor-based scores [43]. Male and female patients were assigned a score of one if they possessed any of the ICD-10-CA codes contained in the factor definition (See Supplementary Table 2); otherwise, they were assigned a zero. These factor-based scores were used to calculate ORs and 95% confidence intervals from a conditional logistic regression for the association between TBI and each factor, adjusting for age, rurality and income quantile in the training dataset first, and then repeated in the testing and validation datasets. Pearson’s correlation matrix was generated for all significant factors in all three datasets to identify groups of male and female patients with similar associative factors, and hierarchical clustering was performed on similar group factors using correlation-based distance. To aid in the visualization of clusters of factors at the event phase, we used heatmaps, where clustering was performed using Ward (minimum variance) linkages. The dendrogram/clustering used to group the factors was based on our previous work [8]. The algorithms for these agglomerative clustering methods have been described elsewhere [44, 45].

To visualize sex-specific differences in the host, agent, and environment factor correlations, raw correlation values were plotted onto heatmaps. To remove noise from weaker correlations, hypothesis tests were conducted using Fisher-transformed correlations to identify a set of significant correlations [46, 47]. All correlations (*r*) were transformed using the following formula:

2 $${r}^{\prime }=\frac{1}{2}\mathit{\ln}\left(\frac{1+r}{1-r}\right)$$

The resulting transformed value *r*′ has variance $\frac{1}{n-3}$ and quickly converges to normal, a property which is useful for conducting z-tests. Three sets of hypotheses tests were conducted to generate heatmaps for males ${\Big(H}_M^0\Big)$, females $\Big({H}_F^0\Big)$, and the differences between both sexes $\Big({H}_{F-M}^0\Big)$:

3 $${H}_M^0:{r}^{\prime }=0$$

4 $${H}_F^0:{r}^{\prime }=0$$

5 $${H}_{F-M}^0:{r}_F^{\prime }-{r}_M^{\prime }=0$$

For the first two hypotheses (3) and (4), the test statistic was calculated as

6 $$z=\frac{r^{\prime }}{\sqrt{\frac{1}{n-3}}}$$

For hypothesis (5), the test statistic was calculated as

7 $$z=\frac{r_F^{\prime }-{r}_M^{\prime }}{\sqrt{\frac{1}{n_F-3}+\frac{1}{n_M-3}}}$$

where the resulting statistic comes from a normal distribution *N*(0, 1) which can be used to calculate *p*-values. Multiple testing correction (i.e., the BY method) was then used as previously described to obtain a set of *p*-values with the FDR controlled at 5%.

Finally, the host, agent, environment-related variables were placed on the association matrix for TBI severity, and the external cause of injury as designated by the CDC [7]. Using the same procedure outlined above, sex-specific heatmaps were generated using Fisher transformation, z-tests, and multiple hypothesis testing correction to control the FDR at 5%. SAS software (SAS Inc., Cary, NC version 9.410) and R (R Foundation for Statistical Computing version 3.4.1.11,) were used for data analyses. Figures were created using R (ComplexHeatmap and Wordcloud, R Foundation for Statistical Computing; www.r-project.org).

## Results

Among the total Ontario population of 12.9 and 14 million in 2008 and 2016, respectively [30], 239,103 unique patients had a TBI-related visit to either an ED or acute care hospital between the years 2007/08 and 2015/16. Every patient with TBI was matched to a patient from the 10% random sample of patients entering an ED or acute care hospital for any reason but TBI; 4100 (1.7%) patients were left unmatched and were removed from further analysis. The final dataset consisted of 235,003 patients; it was split randomly into testing (25%; *n* = 58,516), validation (25%; *n* = 58,798), and training (50%; *n* = 117,689) datasets.

### External causes of TBI

Of the 58,516 patients in the testing dataset, 57% were male and 43% were female patients. The most common TBI mechanisms were falls (*n* = 26,480 [45%]; male: *n* = 13,400 [40%]; female: *n* = 13,080 [52%]) and being struck by/against an object (*n* = 20,845 [36%]; male: *n* = 13,370 [40%]; female: *n* = 7475 [30%]). Ten percent were sustained in MVC, equally distributed among the sexes. Of all injuries, 25% were sports-related, affecting 29% of the male and 19% of the female patients, and assaults accounted for 7% of TBIs, affecting 10% of the male and 4% of the female patients. Injury severity was not established in 25,036 [43%] patients; among these, most cases were coded as concussions without a specified length of unconsciousness (ICD-10-CA code S06.0) (Table 1).

**Table 1:** Characteristics of patients with a first traumatic brain injury-related visit in the emergency department or acute care and matched reference patients

Variables	Patients with TBI (***n*** = 58,516)		Reference patients (***n*** = 58,516)	
	Female (*n* = 25,137; 43%)	Male (*n* = 33,379; 57%)	Female (*n* = 25,137; 43%)	Male (*n* = 33,379; 57%)
Sociodemographic characteristics				
*Age at injury (years)*	39.41 (26.3)	33.84 (24.3)	39.41 (26.3)	33.85 (24.3)
*Income quintile*				
Q1 (lowest)	4868 (19)	6597 (20)	4868 (19)	6597 (20)
Q2	4895 (19)	6645 (20)	4895 (19)	6645 (20)
Q3	4956 (20)	6538 (20)	4956 (20)	6538 (20)
Q4	5269 (21)	6913 (21)	5269 (21)	6913 (21)
Q5 (highest)	5149 (20)	6686 (20)	5149 (20)	6686 (20)
*Rurality* (yes)	3842 (15)	5242 (16)	3842 (15)	5242 (16)
TBI-related characteristics				
*TBI main diagnosis*	22,163 (88)	29,542 (89)	NA	NA
*Injury severity*				
Unspecified	12,075 (48)	12,961 (39)	NA	NA
Mild	8260 (33)	12,201 (37)	NA	NA
Moderate	682 (3)	1484 (4)	NA	NA
Severe	4120 (16)	6733 (20)	NA	NA
Type of first healthcare entry				
Emergency Department	21,284 (85)	26,858 (80)	NA	NA
Acute Care	1606 (6)	2541 (8)	NA	NA
Emergency & Acute^a^	2247 (9)	3980 (12)	NA	NA
External causes of injury, by CDC^b^				
Sports injury	4668 (19)	9804 (29)	NA	NA
Assault	928 (4)	3431 (10)	NA	NA
Falls	13,080 (52)	13,400 (40)	NA	NA
Motor vehicle collisions	2587 (10)	3221 (10)	NA	NA
Struck by/against	7475 (30)	13,370 (40)	NA	NA
Other	2519 (10)	4272 (13)	NA	NA
Missing	44 (0)	122 (0)	NA	NA

*Abbreviations: Q* Quantile, *NA* Not applicable, *TBI* Traumatic brain injury, *SD* Standard deviation. Data presented as n (%) or mean (standard deviation). ^a^A patient had a transfer to either location on the same day. ^b^A patient may have several designations (i.e., sports injury and struct by/against an object)

### Sex-specific health status of the host, agent, and environment at the TBI event phase

**Fig. 1:**
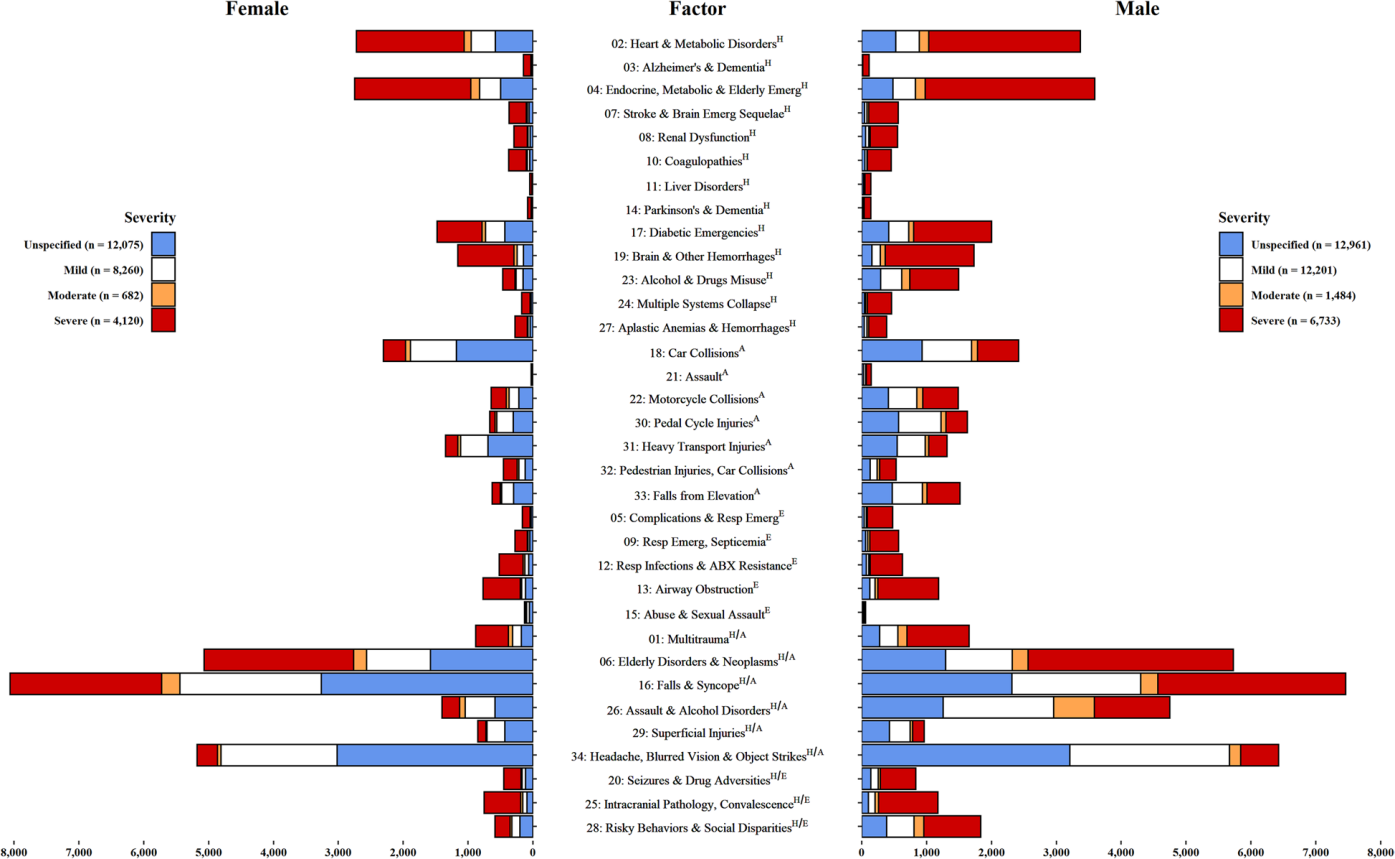
Host (H), agent (A), environment (E) and intertwined (H/A and H/E) factors in male and female patients with TBI in Ontario, Canada 2005/06–2015/16. Data are shown by injury severity

From all possible 2600 codes classifying the TBI and reference patients’ health conditions at the ED or acute care settings at the event phase on which matched McNemar tests were performed, 273 significant associations (i.e., had an OR greater than one) were found after BY correction, of which 226 (83%) were internally validated. Of the 226 codes included in the factor analysis, 164 (73%) unique codes met the factor loading cut-off of 0.2 (Supplementary Table 2). Thirty-four factors were selected based on their interpretability and the breakpoints on the scree plots from further analyses (Table 2 and Fig. 1). For details on frequencies, ORs, and factor loadings of codes that met the factor analysis cut-off and rationale for Host, Agent, and environment designations, please see Supplementary Table 3.

**Table 2:** Characteristics of male and female patients with TBI by injury severity

VARIABLES	FEMALES, by injury severity (25,137, %)				MALES, by injury severity (33,379, %)			
	Unspecified (12,075, 48.0)	Mild (8260, 32.9)	Moderate (682, 2.7)	Severe (4120, 16.4)	Unspecified (12,961, 38.8)	Mild (12,201, 36.6)	Moderate (1484, 4.5)	Severe (6733, 20.2)
Sociodemographic characteristics [H = host; E = Environment]								
Age at first TBI, years old ^H^								
Mean (SD)	33.51 (22.3)	33.1 (22.9)	55.5 (25.7)	66.7 (25.0)	27.8 (20.3)	26.8 (19.5)	40.7 (21.6)	56.7 (25.4)
Median (Q1-Q3)	27 (16–49)	25 (15–49)	60 (33–78)	76 (55–85)	20 (13–40)	19 (13–37)	36 (23–56)	62 (36–79)
Income quantile Q1 (lowest)^H^	2255 (18.7)	1567 (19.0)	137 (20.1)	909 (22.1)	2409 (18.6)	2307 (18.9)	389 (26.2)	1492 (22.2)
Q2	2334 (19.3)	1568 (19.0)	153 (22.4)	840 (20.4)	2473 (19.1)	2396 (19.6)	300 (20.2)	1476 (21.9)
Q3	2399 (19.9)	1616 (19.6)	126 (18.5)	815 (19.8)	2572 (19.8)	2360 (19.3)	256 (17.3)	1350 (20.1)
Q4	2645 (21.9)	1725 (20.9)	133 (19.5)	766 (18.6)	2784 (21.5)	2596 (21.3)	272 (18.3)	1261 (18.7)
Q5 (highest)	2442 (20.2)	1784 (21.6)	133 (19.5)	790 (19.2)	2723 (21.0)	2542 (20.8)	267 (18.0)	1154 (17.1)
Rurality (yes)^E^	2000 (16.6)	1269 (15.4)	78 (11.4)	495 (12.0)	2213 (17.1)	1924 (15.8)	192 (12.9)	913 (13.6)
Factors [A = agent &vector; H = host; E = Environment]								
1. Multitrauma^H/A^	178 (1.5)	134 (1.6)	66 (9.7)	504 (12.2)	274 (2.1)	282 (2.3)	141 (9.5)	956 (14.2)
2. Heart & Metabolic Disorders^H^	577 (4.8)	374 (4.5)	112 (16.4)	1660 (40.3)	525 (4.1)	365 (3.00)	143 (9.6)	2339 (34.7)
3. Alzheimer’s & Dementia^H^	17 (0.1)	14 (0.2)	NA (NA)	115 (2.8)	13 (0.1)	NA (NA)	NA (NA)	93 (1.4)
4. Endocrine, Metabolic & Elderly Emergencies^H^	497 (4.1)	324 (3.9)	135 (19.8)	1791 (43.5)	480 (3.7)	347 (2.8)	154 (10.4)	2610 (38.8)
5. Complications & Resp Emergencies^E^	28 (0.2)	15 (0.2)	NA (NA)	118 (2.9)	39 (0.3)	29 (0.2)	21 (1.4)	385 (5.7)
6. Elderly Disorders, Neoplasms & Falls^H/A^	1580 (13.1)	985 (11.9)	200 (29.3)	2302 (55.9)	1291 (10.0)	1032 (8.5)	241 (16.2)	3162 (47.0)
7. Stroke & Brain Emerg Sequelae^H^	62 (0.5)	25 (0.3)	15 (2.2)	265 (6.4)	45 (0.4)	39 (0.3)	23 (1.6)	455 (6.8)
8. Renal Dysfunction^H^	46 (0.4)	31 (0.4)	8 (1.2)	209 (5.1)	62 (0.5)	45 (0.4)	24 (1.6)	420 (6.2)
9. Resp Emerg, Septicemia^E^	48 (0.4)	27 (0.3)	10 (1.5)	189 (4.6)	60 (0.5)	33 (0.3)	30 (2.0)	447 (6.6)
10. Coagulopathies^H^	50 (0.4)	39 (0.5)	15 (2.2)	268 (6.5)	50 (0.4)	29 (0.2)	10 (0.7)	365 (5.4)
11. Liver Disorders^H^	8 (0.1)	NA (NA)	NA (NA)	35 (0.9)	26 (0.2)	21 (0.2)	NA (NA)	95 (1.4)
12. Resp Infections & ABX Resistance^E^	64 (0.5)	63 (0.8)	24 (3.5)	369 (9.0)	68 (0.5)	38 (0.3)	26 (1.8)	495 (7.4)
13. Airway Obstruction^E^	113 (0.9)	59 (0.7)	25 (3.7)	572 (13.9)	126 (1.0)	78 (0.6)	46 (3.1)	937 (13.9)
14. Parkinson’s & Dementia^H^	14 (0.1)	9 (0.1)	NA (NA)	52 (1.3)	20 (0.2)	10 (0.1)	NA (NA)	104 (1.5)
15. Abuse & Sexual Assault^E^	54 (0.5)	45 (0.5)	11 (1.6)	20 (0.5)	16 (0.1)	23 (0.2)	NA (NA)	22 (0.3)
16. Falls & Syncope^HA^	3260 (27.0)	2180 (26.4)	280 (41.1)	2339 (56.8)	2317 (17.9)	1981 (16.2)	267 (18.0)	2892 (43.0)
17. Diabetic Emergencies^H^	435 (3.6)	296 (3.6)	56 (8.2)	691 (16.8)	416 (3.2)	309 (2.5)	74 (5.0)	1202 (17.9)
18. Car Collision^A^	1180 (9.8)	706 (8.6)	76 (11.1)	344 (8.4)	932 (7.2)	760 (6.2)	95 (6.4)	630 (9.4)
19. Brain & Other Hemorrhages^H^	148 (1.2)	96 (1.2)	50 (7.3)	864 (21.0)	159 (1.2)	129 (1.1)	74 (5.0)	1367 (20.3)
20. Seizures & Drug Adversities^H/E^	114 (0.9)	55 (0.7)	13 (1.9)	269 (6.5)	142 (1.1)	115 (0.9)	31 (2.1)	545 (8.1)
21. Assault^A^	NA (NA)	8 (0.1)	NA (NA)	16 (0.4)	25 (0.2)	36 (0.3)	11 (0.7)	75 (1.1)
22. Motorcycle Collision^A^	218 (1.8)	152 (1.8)	40 (5.9)	234 (5.7)	414 (3.2)	437 (3.6)	90 (6.1)	548 (8.1)
23. Alcohol & Drugs Misuse^H^	152 (1.3)	106 (1.3)	18 (2.6)	192 (4.7)	290 (2.2)	325 (2.7)	126 (8.5)	751 (11.2)
24. Multiple Systems Collapse^H^	26 (0.2)	8 (0.1)	10 (1.5)	127 (3.1)	42 (0.3)	19 (0.2)	28 (1.9)	369 (5.5)
25. Intracranial Pathology, Convalescence^H/E^	90 (0.8)	66 (0.8)	34 (5.0)	561 (13.6)	103 (0.8)	101 (0.8)	56 (3.8)	914 (13.6)
26. Assault & Alcohol Disorders^H/A^	586 (4.9)	456 (5.5)	88 (12.9)	271 (6.6)	1256 (9.7)	1703 (14.0)	628 (42.3)	1163 (17.3)
27. Aplastic Anemias & Hemorrhages^H^	45 (0.4)	33 (0.4)	10 (1.5)	188 (4.6)	37 (0.3)	43 (0.4)	27 (1.8)	276 (4.1)
28. Risky Behaviors, Drug Disorders & Social Disparities ^H/E^	199 (1.7)	128 (1.6)	26 (3.8)	229 (5.6)	385 (3.0)	421 (3.5)	150 (10.1)	879 (13.1)
29. Superficial Injuries^H/A^	432 (3.6)	277 (3.4)	16 (2.4)	123 (3.0)	425 (3.3)	319 (2.6)	40 (2.7)	181 (2.7)
30. Pedal cycle Injuries^A^	305 (2.5)	255 (3.1)	27 (4.0)	76 (1.8)	569 (4.4)	656 (5.4)	76 (5.1)	328 (4.9)
31. Heavy Transport Injuries^A^	693 (5.7)	419 (5.1)	46 (6.7)	189 (4.6)	546 (4.2)	432 (3.5)	53 (3.6)	283 (4.2)
32. Pedestrian Injuries, Car Collision^A^	117 (1.0)	102 (1.2)	26 (3.8)	207 (5.0)	128 (1.0)	108 (0.9)	42 (2.8)	252 (3.7)
33. Falls from Elevation^A^	297 (2.5)	186 (2.3)	22 (3.2)	125 (3.0)	471 (3.6)	467 (3.8)	66 (4.5)	513 (7.6)
34. Headache, Blurred Vision & Object Strikes^H/A^	3020 (25.0)	1787 (21.6)	57 (8.4)	314 (7.6)	3208 (24.8)	2459 (20.2)	175 (11.8)	584 (8.7)

*Abbreviations: ABX* Antibiotic, *A* Agent, *E* Environment, *H* Host. *NA* Not available; frequencies of less than 6 hidden to protect confidentiality

For female patients, the host factors with the highest representation within severe TBIs were Heart and Metabolic Disorders (Factor 2), Metabolic and Elderly Emergencies (Factor 4), and Parkinson’s and Dementia (Factor 14); while the lowest share occurred in Headaches, Blurred Vision and Objects Strikes (Factor 34). The agent (i.e., elements that are necessary for the initiation of the pathological process of a TBI by the external force to the head) was most often Falls and Syncope (Factor 16), Car (Factor 18), and Heavy Transport Accidents (Factors 31), in which less severe injuries were prevalent (Fig. 1). Table 2 presents the frequencies, ORs, and ICD-10 codes composing each of the 34 factors. Supplementary Table 3 presents factor loading and a detailed description of each factor.

### Sex-specific distribution of the host, agent, and environment at the TBI event phase

**Fig. 2:**
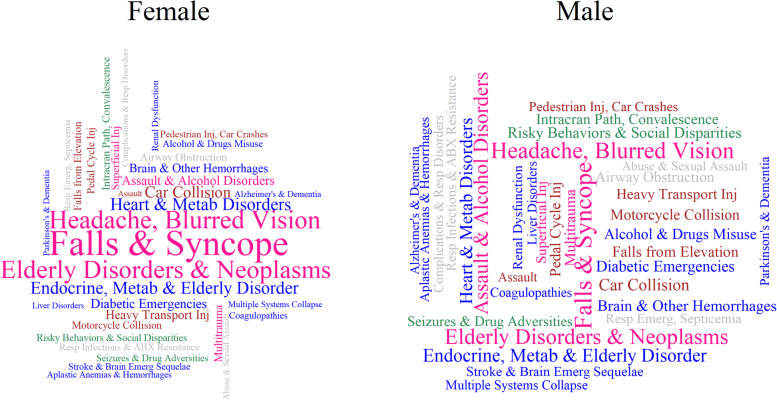
Wordcloud of factors, by proportion, in male and female patients. The word size is relational to the proportion that the factor appears during the current injury event window in female (*N* = 25,137) or male (*N* = 33,379) patients with traumatic brain injury. The colours represent classifications of the Haddon Matrix as follows: host = “blue”; agent = “brown”; environment = “grey”; host/agent = “deep pink”; host/environment = “sea green”

**Fig. 3:**
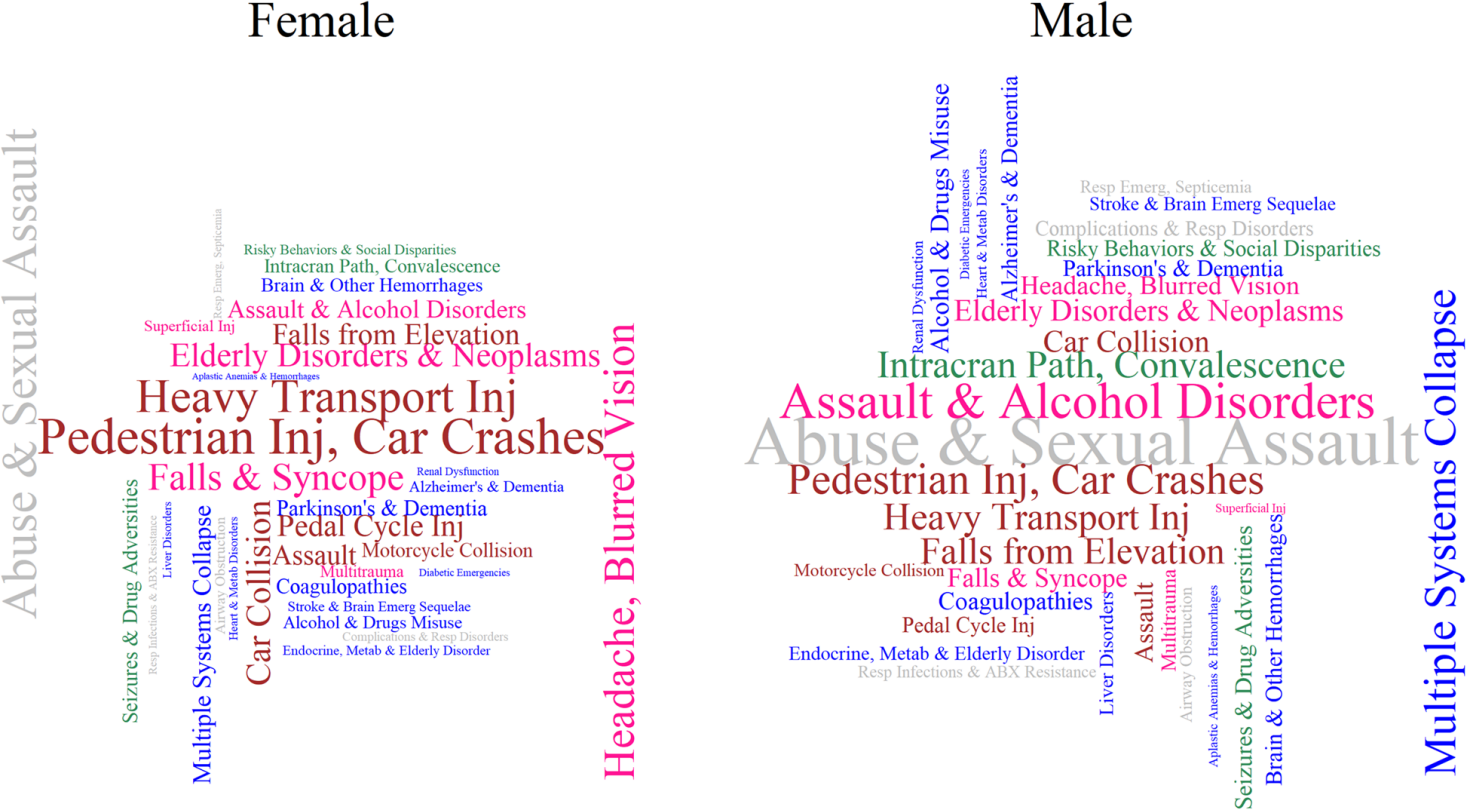
Wordcloud of factors by the magnitude of effect size. The word size is proportional to the magnitude of the Odds Ratio (OR), measuring the association between possessing the factor during the current injury event window. The ORs were calculated using logistic regression in the testing dataset, controlled for rurality, age, place of residence, and income quintile. The colors represent classifications of the Haddon Matrix as follows: host = “blue”; agent = “brown”; environment = “grey”; host/agent = “deep pink”; host/environment = “sea green”

When factors were sorted by their proportion values in the TBI event, Factor 16 (Falls and Syncope), Factor 34 (Headache, Blurred Vision), and Factor 6 (Elderly Disorders & Neoplasms), were the three factors with the highest proportion in both sexes (Fig. 2). When these factors were sorted by their effect size (OR), Abuse and Sexual Assault (Factor 15) and Assault and Alcohol disorders (Factor 26) held the strongest associations with TBI in male patients. In females, the top two factors associated with TBI were injuries induced by transport (e.g., Pedestrian Injuries and Heavy Transport Injuries, Factor 31 and Factor 32, respectively) (Fig. 3).

### Heatmaps connecting the host, agent, and environment

Sex-specific heatmaps of the clusters connecting the host, agent, and environment factors at the TBI event phase, FDR-adjusted, are presented in Supplementary Figs. 1 and 2. These heatmaps were generated and compared between the training, validation, and testing datasets to confirm observed patterns. In both male and female patients, three distinct clusters of associations were observed: Cluster A: Advanced Age-related Brain Pathology; Cluster B: Multiple Body System Pathology; and Cluster C: Young Age-related Concerns.

**Fig. 4:**
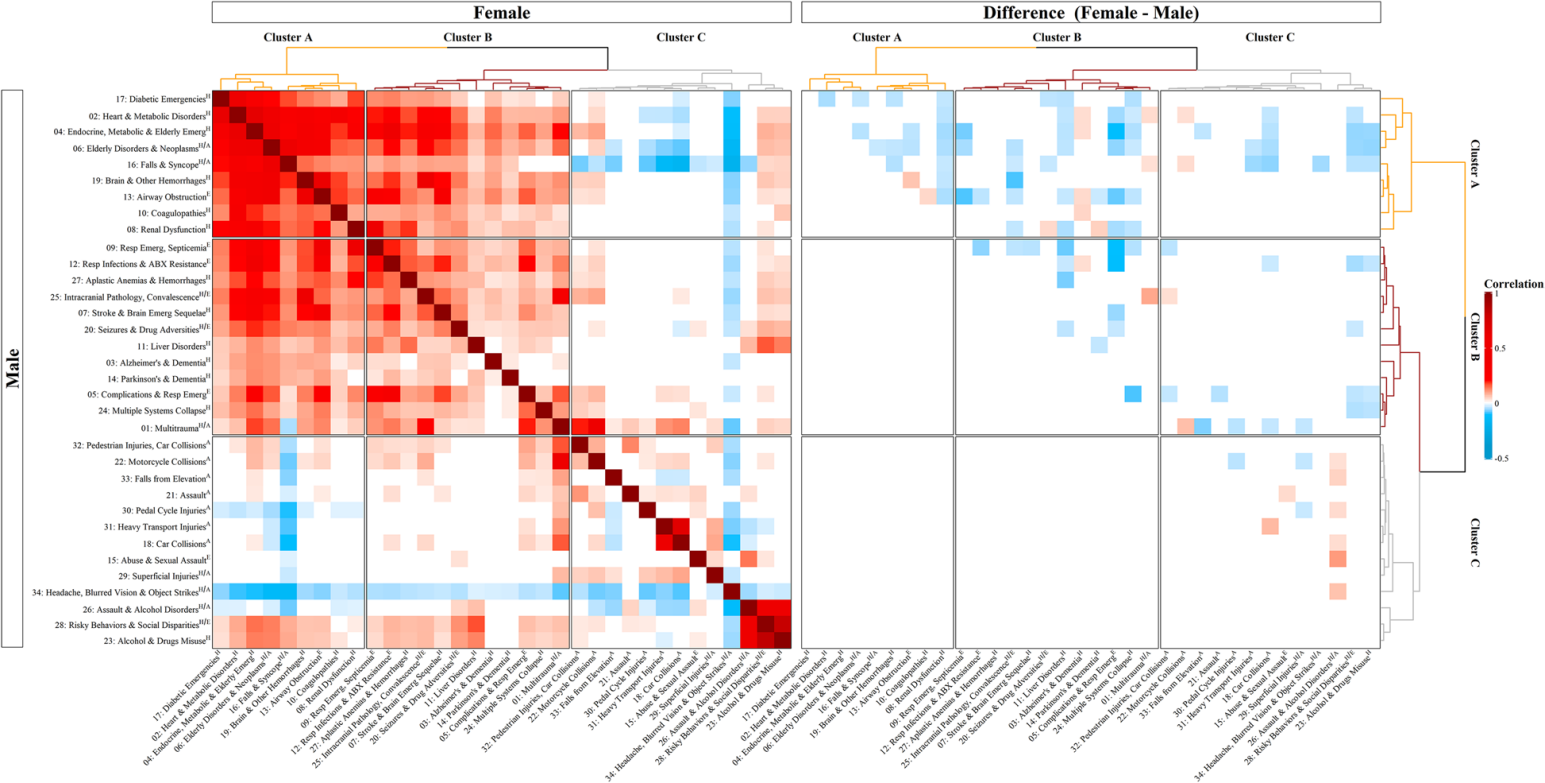
The Host, agent, and environment factors correlations (a) and differences between the sexes (female-male) (**b**). In (**a**), a positive association is coded in red, and a negative association is coded in blue. In the heatmap reporting sex differences (**b**), the red color indicates that if the correlation in a sex-specific heatmap was positive (i.e., red color), females have a stronger positive correlation between those two variables than males, and if it was negative (blue color), females have a weaker correlation than males. The blue color in (**b**) indicates that if the original correlation was positive, males have a stronger positive correlation between those two factors, and if negative, males have a weaker correlation than females

Each of these clusters composed the host, agent, and environment factors. Of the nine factors composing Cluster A (Advanced Age-related Brain Pathology), seven (78%) were depicting the host. In contrast, of 13 factors composed Cluster C (Young Age-related Concerns), nine (69%) were depicting agents. Cluster B (Multiple Body System Pathology) was uniting the host and environment factors equally (Fig. 4). The most discrete differences between male and female patients were observed within Cluster C (Young Age-related Concerns), in the Assault & Alcohol Disorders (Factor 26) and Abuse & Sexual Assault (Factor 15), followed by Cluster A (Advanced Age-related Brain Pathology) in the Brain and Other Hemorrhages (Factor 19) and Airway Obstruction (Factor 13).

### Host, agent, and environment in external cause of injury and TBI severity

**Fig. 5:**
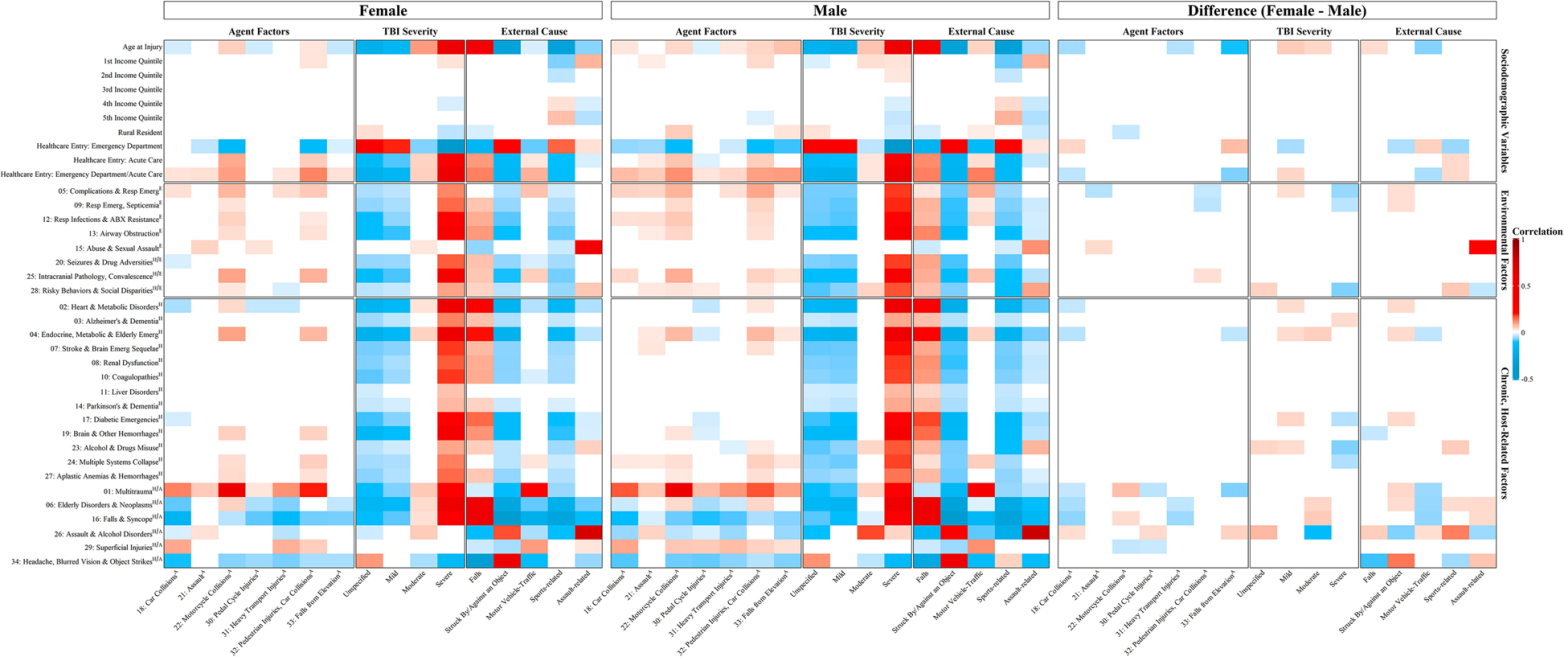
Mapping the Host, Agent, and environment factors on the external cause of injury and TBI severity (**a**) and differences between the sexes (female-male) (**b**). In (**a**), a positive association is presented in red, and a negative association is presented in blue. In the heatmap reporting sex differences (**b**), red colour indicates that if the correlation in a sex-specific heatmap was positive (i.e., red colour), females have a stronger positive correlation between those two variables than males, and if it was negative (blue colour), females have a weaker correlation than males. The blue colour in (**b**) indicates that if the original correlation was positive, males have a stronger positive correlation between those two factors, and if negative, males have a weaker correlation than females

Many of the factors depicting the host and host/environment showed a significant association with external cause of injury and TBI severity. In both male and female patients, falls had a strong positive correlation with chronic disorders within the host, including Heart and Metabolic Disorders (Factor 2), Diabetic, Endocrine, Metabolic, and Elderly Emergencies (Factors 4 and 17). These disorders were also positively associated with severe TBI and increasing age and negatively associated with Abuse and Sexual Assault (Factor 15) and rural residence (Fig. 5). When comparing the sexes, the strength of the association with increasing age was more profoundly seen in female as compared to male patients. Struck by or against an object showed negative associations with age and chronic disorders of the host; the strength was weaker for male patients as compared to female patients.

Motor vehicle collisions were positively correlated with Multisystem Collapse (Factor 24); Intracranial Pathology and Convalescence (Factor 25); and Complications and Respiratory emergencies (Factor 5) in both sexes with an equal strength of association and shared links to severe TBI. In both sexes, sports related TBIs showed strong positive associations with higher income level and negative associations with chronic disorders of the host and adverse social environment. The associations were stronger for female patients as compared to male patients in Alcohol and Drug Misuse (Factor 23) and Risky Behaviours and Social Disparities (Factor 28). Notably, assault as an external cause of injury highlighted strong positive associations with Alcohol and Drug Misuse (Factor 23), and Risky Behaviours and Social Disparities (Factor 28), as well as Abuse and Sexual Assault (Factor 15), with much stronger effect seen in male patients as compared to female patients.

When we analysed the agent factors (Fig. 5), sex-specific patterns in the host were observed. Car Collisions and Falls from Elevation (Factors 18 and 33) were positively associated with Multiple System Collapse and Intracranial Pathology and Convalescence within the Host (Factors 24 and Factor 25) in male but not female patients. The agent Assault (Factor 21) was positively associated with Abuse and Sexual Assault (Factor 15) in female but not male patients. When sex differences were analysed, it appeared that in male patients, the association between the agent factor Assault (Factor 21) and the environment factor Abuse and Sexual Assault (Factor 21) was stronger than in female patients. Other noteworthy observation included the direction of associations between increasing age and Falls from Elevation (Factor 33), positive for male patients and negative for female patients.

All reported results were confirmed in the training and validation datasets, and they were confirmed to be robust (Supplementary Figs. 3 and 4).

## Discussion

Our research is the first, to the best of our knowledge, to use the computational and visual techniques and the Haddon Matrix to analyze data from the injury event of patients entering ED or acute care facilities with a TBI-related diagnosis code in Ontario, Canada, over a period of 10 years. We present previously known and well-established associations, which support the theoretical validity of the approach, as well as sex-specific associations that are not initially intuitive, without any predetermined human bias. The results highlight differences between male and female patients with TBI at the injury event in the characteristics of the host, the agent, and the environment. These differences require further investigation for their implications to be established more precisely, as they undoubtedly reflect biological (sex) vulnerability (e.g., probability of sustaining a TBI in falls due to biological differences) and sociocultural (gender) vulnerability (e.g., probability of TBI due to assault), both of which have implications in clinical and preventive contexts, and for TBI surveillance. We also corroborated our prior hypotheses on the dynamics of the TBI-comorbidity relationship at the injury event and its implications for external causes of injury and TBI severity. We issued an exhaustive effort to describe unique aspects of sex differences of the host at the injury event, as well as differences in exposure to adverse social and environmental circumstances, which can be a function of gender, rooted in socially determined expectations and behaviors ascribed on the basis of biological sex [48].

### The host: differences between male and female patients

The host’s biological, psychosocial, and behavioral characteristics have been the primary targets of TBI research, identifying high risk groups for primary, secondary, and tertiary preventive initiatives [49–51]. Several prior studies reporting on in-hospital TBI outcomes found more severe brain pathology in males than in females, irrespective of age [4, 5, 52]. Our results add to the findings from previous research, highlighting the greater frequency of severe TBI in male patients as compared to female patients. Our results also provide insight into the potential underpinnings of this difference between the sexes. For instance, the strength of the association between Multiple System Collapse, in-hospital Diabetic and Respiratory Emergencies, Septicemia, and severe TBI was stronger in male patients as compared to female patients, which may point to their more pronounced physiological dysregulation in response to TBI and biological vulnerability to early adverse outcomes [5].

Marked differences between male and female patients at the TBI event have been identified previously in diseases affecting the cardiovascular, endocrine, and metabolic systems [53], and were likewise captured in this study in Factors 2, 4, 17, and 19. These factors are implicated in the risk of Syncope and Falls (Factor 16) and severe TBI and were more profoundly expressed in female patients than male patients in this study. Since the severity of TBI is assigned according to clinical signs and symptoms [54], increased awareness among knowledge users of the influence of these differences when assessing TBI severity is important [55, 56]. Sex differences in alcohol disorders at the injury event, highlighted in this study, likely contribute to injury severity designations. Substances that cross the blood-brain barrier affect brain function and cognition, impact depth and duration of coma following injury and alter indices of TBI severity. The most widely used measure of consciousness and thereby injury severity post-TBI is the GCS, where coma is assessed by ability to open the eyes, obey commands, and/or utter understandable words, each of which can be affected by alcohol, confounding GCS assessment in trauma patients and possibly impacting assessment of acute care performance in male patients to a greater extent than in female patients [57].

Another finding that warrants discussion is the relationship between abuse, sexual assault, and TBI. It was observed that Factor 15 (Abuse and Sexual Assault) is overrepresented in TBI patients as compared to the reference population. This overrepresentation is particularly pronounced in females, which is consistent with results from previous research. [58, 59]. However, the stronger association between Factor 15 (Abuse and Sexual Assault) and TBI observed in male patients as compared to female patients may suggest that abuse and sexual assault are more likely to go undetected in male patients, as suggested by research from other settings [60, 61]. This is not to say that sexual assault in female patients is necessarily less challenging to capture but that the collection of abuse and sexual assault data in male patients with TBI should be given appropriate consideration given the likely impact of gender norms, expectations, and expressions on reporting in this group [62–64]. Furthermore, the codes that loaded on Factor 15 (T74, Y07, Y05, Y06) point to the complexity of the social environment of a TBI patient and highlighting the importance of providing customized assessment and care to a patient in TBI management.

### The agent: differences between male and female patients

The fundamental tasks in injury control are to prevent the agent from reaching people in a magnitude that exceeds injury thresholds and to minimize the consequences of the injury [21, 22]. The ‘agent’ with respect to the injury event includes everything that determines whether a TBI results from the injury. We studied implications of agent in falls, strikes by/against an object, MVCs, sports injuries, and assaults. A note-worthy potential contribution to the evolution of TBI surveillance is our data-driven confirmation that the host could also be the agent, as in the case of a person with syncope who falls (Factors 6 and 16) or a person under the influence of alcohol who is assaulted (Factor 23). The role of medication management requires ongoing attention in light of the sex differences that we observed in abnormalities of balance and gait and spontaneous postural sway due to changes in neuromuscular activity, a key physiological system that facilitates and controls movement and stability of the person [65–67].

### The environment: differences between male and female patients

In the present work, we characterized the environment of male and female patients at the TBI event. We found that sustaining TBI in a motorcycle collision (Factor 22) was positively and strongly correlated with living in rural areas in male but not female patients; these results are consistent with those of other studies [68–70]. The sex differences found in socioeconomic status associated with rural community suggests that TBI is subject to a social gradient and impacted by geographical, cultural, and personal values as well as residents’ economic and health status [5]. With more refined data on social standing, future data-driven studies may be able to pinpoint the social equity parameters that underlie the geographic variation in TBI [71].

### External cause and TBI severity

External causes of morbidity and mortality codes classify many dimensions of the cause of injury in a single ICD code [72, 73]. The US CDC external causes of injury codes include the dimensions of intent (i.e., assault and unintentional injuries), and cause (falls, struck by/against an object) [7, 34]. The APHEO, when categorizing injuries, adds circumstance of injury (i.e., sport-related injuries) [35]. Our findings, therefore, provide more detailed injury classification schemes as compared to external course codes of injury alone, providing more precision with respect to sex differences in trauma [74, 75], and the mechanism of injury, the circumstances, and the agent involved.

Our data-driven research contributes to the discussion on the clinical definition of TBI severity for the purposes of surveillance, specifically in monitoring sex-specific subgroups by intent of injury (e.g., assault), mechanism of injury (e.g., falls), and circumstance of injury (e.g., struck by/against and object) during a specified activity or place (e.g., in a car when driving machinery, in a field when playing sports). While previous research on TBI severity focused on injury events assumed to be independent of the agent and environment, our results show that the Haddon Matrix designations are not independently contributing to TBI severity but are uniformly present at the injury event and correlate with one another in a sex-specific manner. It follows that if the host, agent, and environment present in a sex-specific manner in a TBI severity, then the workup for designating TBI severity should incorporate the aforementioned triad.

There are limitations to consider when discussing the results of our research. The first limitation concerns the reliability of administrative healthcare data used, and the second concerns complexity of the clustering technique. Despite the population-wide universal health coverage in Ontario, which strives to provide equal access, and the reliability/validity of ICES data on ED and acute care visits [76–78], there may remain uncovered variation between the sexes due to differences in help-seeking behaviors. This may be especially true for certain external causes of TBI, for example, assault related TBI. Sex differences in reporting TBI are well documented in sports and other contexts [79–81]. In an effort to mitigate this issue, we internally validated the results that emerged in the testing dataset using the training and validation datasets and detailed these results in the supplementary material of the manuscript.

The data analyzed in this work concerned the TBI event window and was used to measure the contributions of the host, agent, and environment at the injury event captured in the ED and acute care hospitals. The clustering technique we utilized offers a sound basis for extending the body of knowledge for the fields of medicine, public health, and injury surveillance, as it offered internally validated hierarchical structures into which new ideas concerning the TBI event window and injury severity in male and female patients can be input. Nevertheless, the data-driven process of discovering interesting and meaningful patterns in data using computational analytics methods is still evolving, and how the new methodology may intersect with existing hypothesis-driven research practices in epidemiological research remains uncertain. It is key that our results are not used to make conclusions about causality, but as new sex-specific hypotheses about complex and non-linear interactions of the host, the agent, and the environment in the TBI event window, which are difficult to disentangle via traditional research methodologies. Follow-up to this work should involve a sex-specific prediction of TBI events and injury severity, establishing projection of resource needs and priorities based on the characteristics of the host within their environment.

### Implications for prevention

The goal of prevention is to protect, promote, and maintain health and well-being and to prevent disease, disability, and death [82–84]. Primary prevention aims to circumvent pathology before it occurs. Secondary prevention entails recognition of pathology at its earliest stages, to stop its progression. Tertiary and quaternary prevention involve treatment directed to prevent complications and minimize disability [84]. Stemming from this research, central prevention ideas encompass the need for balance training [85] and addressing cardiovascular syncope [86] in the elderly to prevent TBI sustained in falls (i.e., Elderly disorders & Neoplasms (Factor 6), associated with Alzheimer’s and Dementia (Factor 1) and Falls and Syncope (Factor 16), and clustering on Advanced Age-related Brain Pathology. Education about alcohol intoxication [87] to prevent assault related TBI is similarly important, associated with Factor 26 Assault and Alcohol disorders and Factor 15 Abuse and Sexual Assault in the Young Age-related Concerns cluster; the latter was expressed to a greater extent in male than in female persons with TBI as compared to a reference cohort. Equally important is regulating the environment through government action on road planning, traffic laws, protective equipment, airbags and seatbelts [88–90] to prevent the agent’s energy from reaching the host in an amount that exceeds the injury threshold in traffic accidents associated with Multitrauma (Factor 1) and severe TBI. The observed sex differences across the agent and external cause of injury highlight risk behaviors that differ between the sexes, potentially steeped in socially-promoted gendered behaviours [91]. While it is extremely challenging to modify behaviors [92], it might be possible to educate the public about the effects of gender at the microgeographic level, comprehensively depicting the injury incident and its consequences for groups at risk [93]. Ideas for secondary, tertiary, and quaternary prevention strategies emerged from the results of this research, including attention to interventions directed on the risk associated with the loss of patient autonomy in severe TBI [94], strongly linked to Elderly Disorders & Neoplasms (Factor 6) in both sexes. There exist experimental therapies that inhibit the release of excitotoxins that play an important role in secondary injury, to attenuate cellular oxidative and metabolic stress [95]. Likewise,deep brain stimulation targeting the ascending reticular activating system and areas of the thalamus has been proposed for promoting the autonomy of vulnerable elderly people with TBI [95, 96]. With the widening temporal agent-host-environment window to pre- and post-injury phases through sex lens, the lines of this study may produce substantial advances in the surveillance and prevention of TBI.

## Conclusions

The study findings support the rationale, feasibility, and benefit of computational and data visualization techniques in examining sex-specific patterns within TBI events shaped by social circumstances, providing an enhanced understanding of complex injury data and room to advance TBI surveillance and care. We uncovered meaningful differences between the sexes in the host, agent, and environment and thus contributed to the existing literature in three significant ways. First, our results support the value of ongoing consideration of the differences between male and female patients across the Haddon matrix dimensions. Second, we found that the host’s health status, the agent and the environment are of great relevance to the designation of injury severity and external cause of injury, providing room to advance TBI surveillance. Finally, this study expands the existing knowledge on the healthcare services needed based on the intent of injury (e.g., assault), nature of injury (e.g., falls), or activity when injured (e.g., sports). Future development and application of data-driven methods will facilitate a better understanding of health and harmful environmental exposures of male and female persons across the lifespan and advance the fields of both precision TBI medicine and injury surveillance.

## Supplementary Information

**Additional file 1: Supplementary Table 1.** Data-driven studies examining TBI event, with results presented by the Haddon Matrix designations. **Supplementary Table 2.** Frequencies, ORs, and factor loadings of codes that met the factor analysis cut-off. **Supplementary Table 3.** Frequencies, ORs, and factor loadings of codes that met the factor analysis cut-off and rationale for Host, Agent, and environment designations. **Supplementary Figure 1.** Heatmap of correlation between factors during the injury event-phase period for patients with TBI in the training dataset. **Supplementary Figure 2.** Heatmap of correlation between factors during the injury event-phase period for patients with TBI in the validation dataset. **Supplementary Figure 3.** Heatmap of correlation between Haddon Matrix-classified factors and external cause as well as TBI severity for patients with TBI in the training set. **Supplementary Figure 4.** Heatmap of correlation between Haddon Matrix-classified factors and external cause as well as TBI severity for patients with TBI in the validation set.

## Data Availability

ICES is an independent, non-profit research institute funded by an annual grant from the Ontario Ministry of Health and Long-Term Care (MOHLTC). As a prescribed entity under Ontario’s privacy legislation, ICES is authorized to collect and use health care data for the purposes of health system analysis, evaluation, and decision support. Secure access to these data is governed by policies and procedures that are approved by the Information and Privacy Commissioner of Ontario. While data sharing agreements prohibit ICES from making the dataset publicly available, access may be granted to those who meet prespecified criteria for confidential access, available at www.ices.on.ca/DAS under accession DAS 2016–257(2018 0970 084 000). The complete dataset creation plan and analytic code remain accessible through authors upon request, understanding that the computer programs could rely upon coding templates or macros that are unique to ICES and are therefore either inaccessible or may require adjustment. All of the computer programs and scripts are available upon request from authors.

## Abbreviations

BY: Benjamini-Yekutieli
CDC: Centers for Disease Control and Prevention
CI: Confidence interval
DAD: Discharge Abstract Database
ED: Emergency department
GCS: Glasgow Coma Scale
FDR: False Discovery Rate
ICD-10: International Statistical Classification of Diseases and Related Health Problems, Tenth Revision
NACRS: National Ambulatory Care Reporting System
OR: Odds ratios
TBI: Traumatic brain injury
US: United States
SD: Standard deviation
SAGER: Sex and Gender Equity in Research
STROBE: The Strengthening the Reporting of Observational Studies in Epidemiology
