# Supplementary material for: Sex-specific analysis of traumatic brain injury events: applying computational and data visualization techniques to inform prevention and management
Source: BMC Med Res Methodol. 2022 Jan 30;22:30. doi: 10.1186/s12874-021-01493-6 (PMC8802441; doi:10.1186/s12874-021-01493-6)

**Supplementary Tables and Figures**

[**Supplementary Table 1**. Data-driven studies examining TBI event, with results presented by the Haddon Matrix designations. 2](#_Toc71104760)

[**Supplementary Table 2**. Frequencies, ORs, and factor loadings of codes that met the factor analysis cut-off. 5](#_Toc71104761)

[**Supplementary Table 3**: Frequencies, ORs, and factor loadings of codes that met the factor analysis cut-off and rationale for Host, Agent, and Environment designations. 10](#_Toc71104762)

[**Supplementary Figure 1**: Heatmap of correlation between factors during the injury event-phase period for patients with TBI in the training dataset. 13](#_Toc71104763)

[**Supplementary Figure 2**: Heatmap of correlation between factors during the injury event-phase period for patients with TBI in the validation dataset. 14](#_Toc71104764)

[**Supplementary Figure 3**: Heatmap of correlation between Haddon Matrix-classified factors and external cause as well as TBI severity for patients with TBI in the training set. 15](#_Toc71104765)

[**Supplementary Figure 4**: Heatmap of correlation between Haddon Matrix-classified factors and external cause as well as TBI severity for patients with TBI in the validation set. 16](#_Toc71104766)

# **Supplementary Table 1**. Data-driven studies examining TBI event, with results presented by the Haddon Matrix designations.

PubMed was searched for works published in the last twenty years using the terms “traumatic brain injury” and “data driven” in October of 2020. Of 211 identified citations, eight cohort studies focused on the TBI event window. Note: Despite our attempts to collect all relevant articles, it is possible that some studies were missed due to indexing failures (not all the main concepts addressed in the articles appear as descriptors) and due to the possible lack of suitable descriptors for representing the concepts of data driven TBI research.

| Author/year | **Matsuo et.al, 2020^1^** | **Raj et al.,**  **2019^2^** | **Rau et al.,**  **2018^3^** | **Mitra et al.,**  **2016^4^** | **Gravesteijn et al., 2020^5^** | **Asgari et al., 2019^6^** | **Folweiler et al., 2020^7^** | **Hernandes Rocha et al., 2019^8^** |
| --- | --- | --- | --- | --- | --- | --- | --- | --- |
| Sample/  setting | 232 out of 268 persons; all ages but<10 y.o.; intensive care unit/Japan | 472 adults  TBI patients (>16 y.o.) admitted to tertiary academic  ICUs (Helsinki University Hospital, Kuopio University Hospital, and  Turku University Hospital/Europe | 1,734 TBI adult patients registered in the Trauma Registry System, Taiwan | 179 TBI patients, Australia | 15 studies (n=1,554) from the IMPACT-II database  (4 observational studies and eleven randomized  controlled trials on moderate to severe TBI), Sweden | 379 TBI  patients Addenbrooke Hospital/Cambridge UK | 213 TBI patients who participated in the Citicoline Brain Injury Treatment Trial (COBRIT) Trial/USA | 3,138 patients from TBI data registry Kilimanjaro Christian Medical Centre in Moshi/ Tanzania |
| TBI definition | Abnormal findings for head CT, Marshal CT classification, GCS, ICP | Via electronic health records (including emergency medical service reports, hospital records, surgical  reports, laboratory reports and picture achieving and communication systems | Abbreviated  Injury Scale (AIS) score ≥ 3 points | T1-weighted MRI  GCS | Not reported | Not reported | T1-weighted MRI  GCS | Not reported |
| Methods | Training data  and test data  Supervised, using Scikit-learn: 9 in total): ridge  regression,  LASSO  regression, random forest, gradient boosting, extra trees, decision  tree, Gaussian naїve Bayes, multi-nomial naїve Bayes, and  support vector machine (SVM) (its kernel consisted of linear, radial  basis function [RBF], polynomial [poly], and sigmoid) | Fully automated and dynamic algorithm; performance evaluated by  area under the receiver operating characteristic  curve (AUC) as a function of time, in order to assess how the algorithms performed at different time  points | Logistic regression [LR], support vector machine [SVM], decision tree [DT], naive  Bayes [NB], and artificial neural networks [ANN] | Network-based statistical analysis  Principal component analysis  and subsequent classification by random forest | Logistic regression (LR), lasso regression, and ridge regression  ML algorithms:  vector machines, random forests, gradient boosting  machines, and artificial neural networks | Unsupervised learning to multidimensional time-series TBI physiology  Hidden Markov model | Unsupervised learning framework  GLRM framework to select the most relevant features during our clustering analysis  Generalized low-rank models | 9 different machine learning techniques.  Bayesian generalized linear model |
| Outcome | In-hospital poor outcome (77.6%)  In-hospital mortality (26.3%) | 30-days mortality in a real-time fashion during intensive care | In-hospital mortality | TBI classification | Prediction of 6-month outcome (mortality and GCS<4) for moderate  and severe TBI | Death  6-month Glasgow Outcome Scale (GOS): severe disability, moderate disability, good outcome | Phenotype-specific differences in long-term functional outcome trajectories via Extended Glasgow Outcome Scale (GOS-E) | Discharge Extended Glasgow Outcome Scale (GOS-E) |
| Predictive parameters studied, by A-H-E | Age ^H^  GCS score ^H^  Abnormal  pupillary response ^H^  SBP^H^  Major extracranial injury ^A^  CT findings ^H^  Routinely collected laboratory values^H^ (glucose,  C-reactive protein [CRP], and fibrin/fibrinogen degradation products  [FDP]) | 13 dynamic features and patient age:  Sex, age^H^  GCS^H^  Motor score^H^  Pupillary light reactivity^H^  Hypoxia^H^  Hypotension^H^  Marshal CT^A^  tSAH on CT^A^  Epidural mass on CT^A^  Glucose^H^  Hb^H^  ICU LOS^E^  Neurosurgical procedure^E^ | Age^H^  Sex^H^  Helmet-wearing  Status^A^  Pre-existed co-morbidities^H^: coronary artery disease (CAD), congestive heart failure  (CHF), cerebral vascular accident (CVA), diabetes mellitus (DM), end-stage renal disease  (ESRD), and hypertension (HTN)  Glasgow coma scale (GCS)^H^  Vital signs ^H^:  temperature, systolic blood pressure (SBP), heart rate (HR), and respiratory rate (RR) | Age and sex ^H^ match with no- injury control | 11 predictors of  the IMPACT laboratory model:  Age^H^  Motor GSC^H^  CT class^A^  Traumatic subarachnoid  Hemorrhage^A^  Epidural hematoma^A^  Hypoxia^H^  Hypotension^H^  Glucose^H^  Sodium^H^  Hemoglobin^H^ | Age^H^  Sex^H^  GCS^H^  ICP^H^  Arterial blood pressure ^H^  Data length^E^  Intracranial pressure^H^  Cerebral perfusion pressure^H^  Compensatory reserve index^H^  Pressure reactivity index^H^ | Age^H^  Race^H^  Ethnicity^H^  Education level^H^  Hematology measures ^H^:  Platelet count Hematocrit  Hemoglobin  Coagulation measures^H^ : Prothrombin time (PT)  PT international normalized ratio (INR)  Blood glucose levels  Mechanism of injury^A^  Midline shift^H^ | Age^H^  Sex ^H^  Vital signs^H^  Mechanism of injury^A^  Surgical intervention^E^ |
| Results, by A-H-E | Ranked as the three most important parameters  Poor outcome:  Age ^H^  Lower GCS^H^  FDP ^H^  Mortality:  FDP^H^  GCS^H^  Abnormal pupillary response^H^ | Three most important parameters  ICP^H^  mean arterial pressure [MAP]^H^  Cerebral perfusion^H^  pressure [CPP] Glasgow Coma Scale [GCS])^H^ | Age^H^  Helmet status^A^  Platelets^H^  DM^H^  SDH^H^  Hct^H^  Temperature^H^ ICH^H^  GCS^H^  Glucose^H^  ISS^A^  End-stage renal disease^H^ | N/A  Pathways associated with  the corpus callosum, the fornix and superior frontal gyri connections | Focus on methods; not results | Good state:  Low ICP  High CPP^H^  Intact  autoregulation^H^  Preserved  compensatory reserve^H^  Poor state:  Higher ICP^H^  Lower CPP^H^  Reduced compensatory reserve^H^  Loss of autoregulation^H^ | 3 different phenotypes observed- different combinations of ^A,H,E^ variables at the baseline | Good outcome:  GCS score^H^ Increasing pulse oximetry^H^  Undergoing TBI surgery ^E^  Poor outcome:  MVC injury ^A^  Increasing Age^H^ |

Abbreviations: CRP=C-reactive protein; CT=computed tomography; DM=diabetes mellitus; GCS=Glasgow coma scale; GLRM= Generalized Low Rank Models; Hct= hematocrit; PT=Prothrombin time; FDP= Fibrin degradation products; ICP=intracranial pressure; ISS=injury severity score; SDH= subdural hematoma; TBI=traumatic brain injury; tSAH= traumatic subarachnoid hemorrhage

**References:**

1. Matsuo K, Aihara H, Nakai T, Morishita A, Tohma Y, Kohmura E. Machine Learning to Predict In-Hospital Morbidity and Mortality after Traumatic Brain Injury. J Neurotrauma. 2020 Jan 1;37(1):202-210. doi: 10.1089/neu.2018.6276. Epub 2019 Sep 18. PMID: 31359814.
2. Raj R, Luostarinen T, Pursiainen E, Posti JP, Takala RSK, Bendel S, Konttila T, Korja M. Machine learning-based dynamic mortality prediction after traumatic brain injury. Sci Rep. 2019 Nov 27;9(1):17672. doi: 10.1038/s41598-019-53889-6. PMID: 31776366; PMCID: PMC6881446.
3. Rau CS, Kuo PJ, Chien PC, Huang CY, Hsieh HY, Hsieh CH. Mortality prediction in patients with isolated moderate and severe traumatic brain injury using machine learning models. PLoS One. 2018 Nov 9;13(11):e0207192. doi: 10.1371/journal.pone.0207192. PMID: 30412613; PMCID: PMC6226171.
4. Mitra J, Shen KK, Ghose S, Bourgeat P, Fripp J, Salvado O, Pannek K, Taylor DJ, Mathias JL, Rose S. Statistical machine learning to identify traumatic brain injury (TBI) from structural disconnections of white matter networks. Neuroimage. 2016 Apr 1;129:247-259. doi: 10.1016/j.neuroimage.2016.01.056. Epub 2016 Jan 28. PMID: 26827816.
5. Gravesteijn BY, Nieboer D, Ercole A, Lingsma HF, Nelson D, van Calster B, Steyerberg EW; CENTER-TBI collaborators. Machine learning algorithms performed no better than regression models for prognostication in traumatic brain injury. J Clin Epidemiol. 2020 Jun;122:95-107. doi: 10.1016/j.jclinepi.2020.03.005. Epub 2020 Mar 20. PMID: 32201256.
6. Asgari S, Adams H, Kasprowicz M, Czosnyka M, Smielewski P, Ercole A. Feasibility of Hidden Markov Models for the Description of Time-Varying Physiologic State After Severe Traumatic Brain Injury. Crit Care Med. 2019 Nov;47(11):e880-e885. doi: 10.1097/CCM.0000000000003966. PMID: 31517697.
7. Hernandes Rocha TA, Elahi C, Cristina da Silva N, Sakita FM, Fuller A, Mmbaga BT, Green EP, Haglund MM, Staton CA, Nickenig Vissoci JR. A traumatic brain injury prognostic model to support in-hospital triage in a low-income country: a machine learning-based approach. J Neurosurg. 2019 May 10:1-9. doi: 10.3171/2019.2.JNS182098. Epub ahead of print. PMID: 31075779.
8. Folweiler KA, Sandsmark DK, Diaz-Arrastia R, Cohen AS, Masino AJ. Unsupervised Machine Learning Reveals Novel Traumatic Brain Injury Patient Phenotypes with Distinct Acute Injury Profiles and Long-Term Outcomes. J Neurotrauma. 2020 Jun 15;37(12):1431-1444. doi: 10.1089/neu.2019.6705. Epub 2020 Mar 11. PMID: 32008422; PMCID: PMC7249479.

# **Supplementary Table 2**. Frequencies, ORs, and factor loadings of codes that met the factor analysis cut-off.

| **Factor** | **Description** | | **Frequency** | | **OR [95% CI]** | **P-value** | **Factor Loading** |
| --- | --- | --- | --- | --- | --- | --- | --- |
|  |  |  | **TBI** | **Reference** |  |  |  |
| **1** | **Trauma: Chest, abdominal, upper and lower extremity injuries** | | **2,535** | **749** | **3.49 [3.21, 3.79]** | **<0.0001** | **-** |
|  | S36 | Injury of intra-abdominal organs | 518 | 18 | 28.78 [17.99, 46.04] |  | 0.66 |
|  | S27 | Injury of other and unspecified intrathoracic organs | 1,088 | 14 | 77.71 [45.87, 131.66] |  | 0.66 |
|  | S37 | Injury of urinary and pelvic organs | 189 | 18 | 10.50 [6.47, 17.03] |  | 0.48 |
|  | T06 | Other injuries involving multiple body regions, not elsewhere classified | 353 | 12 | 29.42 [16.55, 52.30] |  | 0.38 |
|  | T79 | Certain early complications of trauma, not elsewhere classified | 209 | 19 | 11.00 [6.88, 17.59] |  | 0.38 |
|  | S42 | Fracture of shoulder and upper arm | 1,074 | 485 | 2.23 [2.00, 2.49] |  | 0.3 |
|  | S26 | Injury of heart | NR | <6 | 25.50 [6.21, 104.74] |  | 0.3 |
|  | S72 | Fracture of femur | 421 | 192 | 2.22 [1.87, 2.64] |  | 0.23 |
| **2** | **Cardiology and Disorders of Metabolism: Cardiac disease and metabolic insufficiencies** | | **6,095** | **3,787** | **1.97 [1.88, 2.07]** | **<0.0001** | **-** |
|  | E78 | Disorders of lipoprotein metabolism and other lipidemias | 478 | 302 | 1.61 [1.39, 1.86] |  | 0.59 |
|  | I10 | Essential (primary) hypertension | 3,283 | 1,870 | 1.97 [1.85, 2.10] |  | 0.54 |
|  | I48 | Atrial fibrillation and flutter | 1,333 | 547 | 2.59 [2.33, 2.87] |  | 0.54 |
|  | Z95 | Presence of cardiac and vascular implants and grafts | 558 | 278 | 2.05 [1.77, 2.38] |  | 0.53 |
|  | I50 | Heart failure | 509 | 403 | 1.28 [1.12, 1.46] |  | 0.44 |
|  | Z92 | Personal history of medical treatment | 646 | 122 | 5.44 [4.47, 6.62] |  | 0.34 |
|  | E03 | Other hypothyroidism | 204 | 131 | 1.57 [1.26, 1.96] |  | 0.3 |
|  | E11 | Type 2 diabetes mellitus | 2,813 | 1,699 | 1.80 [1.69, 1.92] |  | 0.23 |
| **3** | **Neurology/Behavioural Neurology: Alzheimer's disease and dementia** | | **267** | **79** | **3.41 [2.65, 4.39]** | **<0.0001** | **-** |
|  | F00 | Dementia in Alzheimer disease | 221 | 62 | 3.56 [2.69, 4.72] |  | 0.95 |
|  | G30 | Alzheimer's disease | 265 | 78 | 3.43 [2.66, 4.42] |  | 0.95 |
| **4** | **Emergency Medicine: Emergencies involving the nervous system, other emergencies** | | **6,338** | **2,873** | **2.85 [2.70, 3.00]** | **<0.0001** | **-** |
|  | E87 | Other disorders of fluid, electrolyte and acid-base balance | 1,064 | 401 | 2.74 [2.44, 3.08] |  | 0.55 |
|  | E83 | Disorders of mineral metabolism | 113 | 54 | 2.09 [1.51, 2.89] |  | 0.43 |
|  | E22 | Hyperfunction of pituitary gland | 114 | 8 | 14.25 [6.96, 29.18] |  | 0.39 |
|  | F05 | Delirium, not induced by alcohol and other psychoactive substances | 689 | 135 | 5.50 [4.54, 6.67] |  | 0.36 |
|  | Z75 | Problems related to medical facilities and other health care | 2,189 | 402 | 6.27 [5.59, 7.03] |  | 0.32 |
|  | N17 | Acute kidney failure | 438 | 259 | 1.72 [1.47, 2.01] |  | 0.31 |
|  | F06 | Other mental disorders due to known physiological condition | 83 | 23 | 3.61 [2.27, 5.73] |  | 0.29 |
|  | B96 | Other bacterial agents as the cause of diseases classified elsewhere | 823 | 377 | 2.27 [2.00, 2.57] |  | 0.23 |
|  | I95 | Hypotension | 425 | 139 | 3.13 [2.58, 3.81] |  | 0.22 |
|  | R41 | Other symptoms and signs involving cognitive functions and awareness | 980 | 185 | 5.52 [4.70, 6.48] |  | 0.21 |
|  | I10 | Essential (primary) hypertension | 3,283 | 1,870 | 1.97 [1.85, 2.10] |  | 0.2 |
| **5** | **Emergency Medicine: Complications of medical procedures** | | **638** | **204** | **3.14 [2.68, 3.67]** | **<0.0001** | **-** |
|  | J95 | Intraoperative and postprocedural complications and disorders of respiratory system, not elsewhere classified | 310 | 35 | 8.86 [6.24, 12.56] |  | 0.75 |
|  | Y84 | Other medical procedures as the cause of abnormal reaction of the patient, or of later complication, without mention of misadventure at the time of the procedure | 453 | 151 | 3.00 [2.50, 3.61] |  | 0.72 |
|  | J15 | Bacterial pneumonia, not elsewhere classified | 176 | 28 | 6.29 [4.22, 9.37] |  | 0.53 |
| **6** | **Neurology/Oncology: Disorders of frailty, neoplastic diseases, and dementia** | | **10,793** | **2,275** | **6.92 [6.54, 7.31]** | **<0.0001** | **-** |
|  | F03 | Unspecified dementia | 634 | 179 | 3.88 [3.26, 4.62] |  | 0.46 |
|  | Z51 | Encounter for other aftercare | 1,269 | 529 | 2.49 [2.25, 2.77] |  | 0.38 |
|  | R29 | Other symptoms and signs involving the nervous and musculoskeletal systems | 644 | 103 | 6.95 [5.57, 8.65] |  | 0.38 |
|  | Z75 | Problems related to medical facilities and other health care | 2,189 | 402 | 6.27 [5.59, 7.03] |  | 0.29 |
|  | W05 | Fall from non-moving wheelchair, nonmotorized scooter and motorized mobility scooter | 440 | 29 | 16.81 [11.32, 24.97] |  | 0.29 |
|  | R26 | Abnormalities of gait and mobility | 173 | 35 | 4.94 [3.44, 7.11] |  | 0.27 |
|  | W19 | Unspecified fall | 6,773 | 884 | 9.00 [8.34, 9.71] |  | 0.26 |
|  | C79 | Secondary malignant neoplasm of other and unspecified sites | 110 | 57 | 1.95 [1.41, 2.69] |  | 0.25 |
|  | F05 | Delirium, not induced by alcohol and other psychoactive substances | 689 | 135 | 5.50 [4.54, 6.67] |  | 0.25 |
|  | Z74 | Problems related to care provider dependency | 89 | 34 | 2.62 [1.76, 3.89] |  | 0.23 |
|  | S72 | Fracture of femur | 421 | 192 | 2.22 [1.87, 2.64] |  | 0.22 |
|  | R41 | Other symptoms and signs involving cognitive functions and awareness | 980 | 185 | 5.52 [4.70, 6.48] |  | 0.22 |
|  | W06 | Fall from bed | 805 | 58 | 14.58 [11.10, 19.16] |  | 0.22 |
| **7** | **Emergency Medicine/Neurology: Stroke** | | **929** | **322** | **2.96 [2.61, 3.37]** | **<0.0001** | **-** |
|  | G81 | Hemiplegia and hemiparesis | 251 | 33 | 7.81 [5.41, 11.29] |  | 0.67 |
|  | R47 | Speech disturbances, not elsewhere classified | 220 | 37 | 6.23 [4.36, 8.90] |  | 0.59 |
|  | I63 | Cerebral infarction | 244 | 120 | 2.04 [1.64, 2.54] |  | 0.5 |
|  | I69 | Sequelae of cerebrovascular disease | 167 | 28 | 6.15 [4.09, 9.23] |  | 0.45 |
|  | R13 | Aphagia and dysphagia | 291 | 166 | 1.76 [1.45, 2.13] |  | 0.31 |
| **8** | **Nephrology: Renal issues** | | **845** | **439** | **2.00 [1.77, 2.25]** | **<0.0001** | **-** |
|  | Z99 | Dependence on enabling machines and devices, not elsewhere classified | 82 | 17 | 4.82 [2.86, 8.13] |  | 0.68 |
|  | N18 | Chronic kidney disease (CKD) | 332 | 177 | 1.91 [1.59, 2.30] |  | 0.66 |
|  | N08 | Glomerular disorders in diseases classified elsewhere | 238 | 77 | 3.15 [2.43, 4.08] |  | 0.64 |
|  | N17 | Acute kidney failure | 438 | 259 | 1.72 [1.47, 2.01] |  | 0.32 |
| **9** | **Nephrology/Respirology/Emergency Medicine: Pulmonary, renal, and systemic emergencies** | | **844** | **378** | **2.32 [2.05, 2.63]** | **<0.0001** | **-** |
|  | J17 | Pneumonia in diseases classified elsewhere | 67 | 17 | 3.94 [2.31, 6.71] |  | 0.79 |
|  | A41 | Other sepsis | 277 | 105 | 2.65 [2.12, 3.33] |  | 0.78 |
|  | N17 | Acute kidney failure | 438 | 259 | 1.72 [1.47, 2.01] |  | 0.26 |
|  | J96 | Respiratory failure, not elsewhere classified | 250 | 61 | 4.20 [3.16, 5.58] |  | 0.23 |
| **10** | **Hematology: Coagulation disorders** | | **826** | **183** | **4.65 [3.95, 5.48]** | **<0.0001** | **-** |
|  | Y44 | Agents primarily affecting blood constituents | 150 | 39 | 3.85 [2.70, 5.47] |  | 0.82 |
|  | D68 | Other coagulation defects | 174 | 44 | 3.95 [2.84, 5.50] |  | 0.8 |
|  | Z92 | Personal history of medical treatment | 646 | 122 | 5.44 [4.47, 6.62] |  | 0.27 |
| **11** | **Gastroenterology: Liver disorders** | | **198** | **71** | **2.79 [2.13, 3.66]** | **<0.0001** | **-** |
|  | K70 | Alcoholic liver disease | 110 | 18 | 6.11 [3.71, 10.06] |  | 0.7 |
|  | R18 | Ascites | 43 | 30 | 1.43 [0.90, 2.28] |  | 0.68 |
|  | K72 | Hepatic failure, not elsewhere classified | 42 | 14 | 3.00 [1.64, 5.49] |  | 0.58 |
|  | B18 | Chronic viral hepatitis | 53 | 20 | 2.65 [1.58, 4.43] |  | 0.27 |
| **12** | **Infectious Diseases: Acute infection and treatment resistance** | | **1,147** | **570** | **2.08 [1.88, 2.31]** | **<0.0001** | **-** |
|  | B95 | Streptococcus, Staphylococcus, and Enterococcus as the cause of diseases classified elsewhere | 238 | 186 | 1.28 [1.06, 1.55] |  | 0.72 |
|  | U82 | Resistance to betalactam antibiotics | 94 | 25 | 3.76 [2.42, 5.84] |  | 0.71 |
|  | B96 | Other bacterial agents as the cause of diseases classified elsewhere | 823 | 377 | 2.27 [2.00, 2.57] |  | 0.34 |
|  | A49 | Bacterial infection of unspecified site | 80 | 37 | 2.19 [1.48, 3.25] |  | 0.33 |
|  | L89 | Pressure ulcer | 143 | 20 | 7.15 [4.48, 11.42] |  | 0.21 |
| **13** | **Emergency Medicine: Foreign body in airway and other** | | **1,956** | **771** | **2.70 [2.48, 2.95]** | **<0.0001** | **-** |
|  | J69 | Pneumonitis due to solids and liquids | 359 | 42 | 8.93 [6.44, 12.37] |  | 0.61 |
|  | W80 | Inhalation and ingestion of other objects causing obstruction of respiratory tract | NR | <6 | 16.00 [3.83, 66.76] |  | 0.52 |
|  | J96 | Respiratory failure, not elsewhere classified | 250 | 61 | 4.20 [3.16, 5.58] |  | 0.39 |
|  | R13 | Aphagia and dysphagia | 291 | 166 | 1.76 [1.45, 2.13] |  | 0.27 |
|  | Z51 | Encounter for other aftercare | 1,269 | 529 | 2.49 [2.25, 2.77] |  | 0.23 |
|  | L89 | Pressure ulcer | 143 | 20 | 7.15 [4.48, 11.42] |  | 0.22 |
| **14** | **Neurology: Parkinson's disease and dementia** | | **221** | **50** | **4.42 [3.25, 6.01]** | **<0.0001** | **-** |
|  | F02 | Dementia in other diseases classified elsewhere | 54 | 8 | 6.75 [3.21, 14.18] |  | 0.79 |
|  | G20 | Parkinson disease | 179 | 38 | 4.71 [3.32, 6.69] |  | 0.75 |
|  | G31 | Other degenerative diseases of nervous system, not elsewhere classified | 41 | 11 | 3.73 [1.92, 7.25] |  | 0.41 |
| **15** | **Trauma: Adult and child abuse, and sexual assault** | | **196** | **15** | **13.07 [7.73, 22.09]** | **<0.0001** | **-** |
|  | T74 | Adult and child abuse, neglect and other maltreatment, confirmed | NR | <6 | 10.00 [3.99, 25.08] |  | 0.8 |
|  | Y07 | Perpetrator* of assault, maltreatment and neglect | 160 | 8 | 20.00 [9.83, 40.68] |  | 0.69 |
|  | Y05 | Sexual assault by bodily force | 17 | 8 | 2.12 [0.92, 4.92] |  | 0.44 |
|  | Y06 | Neglect and abandonment | NR | <6 | NA [NA, NA] |  | 0.22 |
| **16** | **Emergency Medicine: Emergencies involving the nervous system** | | **15,516** | **3,106** | **6.79 [6.48, 7.10]** | **<0.0001** | **-** |
|  | R55 | Syncope and collapse | 1,368 | 737 | 1.88 [1.72, 2.06] |  | 0.59 |
|  | W18 | Other slipping, tripping and stumbling and falls | 4,547 | 486 | 10.29 [9.33, 11.36] |  | 0.58 |
|  | W19 | Unspecified fall | 6,773 | 884 | 9.00 [8.34, 9.71] |  | 0.38 |
|  | W01 | Fall on same level from slipping, tripping and stumbling | 5,732 | 1,016 | 6.17 [5.75, 6.62] |  | 0.23 |
|  | I95 | Hypotension | 425 | 139 | 3.13 [2.58, 3.81] |  | 0.22 |
| **17** | **Endocrinology: Diabetes and diabetic consequences** | | **3,479** | **2,426** | **1.54 [1.45, 1.63]** | **<0.0001** | **-** |
|  | E11 | Type 2 diabetes mellitus | 2,813 | 1,699 | 1.80 [1.69, 1.92] |  | 0.61 |
|  | E14 | Unspecified diabetes mellitus | 1,090 | 858 | 1.29 [1.17, 1.41] |  | 0.57 |
|  | R73 | Elevated blood glucose level | 209 | 139 | 1.51 [1.22, 1.88] |  | 0.52 |
|  | G63 | Polyneuropathy in diseases classified elsewhere | 40 | 13 | 3.08 [1.65, 5.75] |  | 0.32 |
|  | N08 | Glomerular disorders in diseases classified elsewhere | 238 | 77 | 3.15 [2.43, 4.08] |  | 0.26 |
| **18** | **Trauma: Car accident injuries** | | **4,723** | **711** | **7.14 [6.58, 7.75]** | **<0.0001** | **-** |
|  | V43 | Car occupant injured in collision with car, pick-up truck or van | 2,298 | 255 | 9.48 [8.30, 10.82] |  | 0.46 |
|  | V49 | Car occupant injured in other and unspecified transport accidents | 627 | 36 | 17.89 [12.72, 25.14] |  | 0.46 |
|  | V48 | Car occupant injured in noncollision transport accident | 783 | 48 | 16.31 [12.19, 21.83] |  | 0.42 |
|  | V89 | Motor- or nonmotor-vehicle accident, type of vehicle unspecified | 443 | 34 | 13.03 [9.19, 18.47] |  | 0.4 |
|  | V47 | Car occupant injured in collision with fixed or stationary object | 562 | 28 | 20.07 [13.73, 29.34] |  | 0.33 |
|  | T14 | Injury of unspecified body region | 289 | 123 | 2.35 [1.90, 2.90] |  | 0.29 |
|  | Z04 | Encounter for examination and observation for other reasons | 419 | 232 | 1.81 [1.54, 2.12] |  | 0.24 |
|  | V58 | Occupant of pick-up truck or van injured in noncollision transport accident | 161 | 10 | 16.10 [8.50, 30.50] |  | 0.21 |
| **19** | **Emergency Medicine: Brain hemorrhage** | | **2,887** | **744** | **4.24 [3.90, 4.62]** | **<0.0001** | **-** |
|  | I61 | Nontraumatic intracerebral hemorrhage | 216 | 17 | 12.71 [7.76, 20.82] |  | 0.43 |
|  | I60 | Nontraumatic subarachnoid hemorrhage | 188 | 15 | 12.53 [7.41, 21.21] |  | 0.39 |
|  | G91 | Hydrocephalus | 108 | 11 | 9.82 [5.28, 18.26] |  | 0.35 |
|  | I62 | Other and unspecified nontraumatic intracranial hemorrhage | 772 | 12 | 64.33 [36.38, 113.78] |  | 0.32 |
|  | C79 | Secondary malignant neoplasm of other and unspecified sites | 110 | 57 | 1.95 [1.41, 2.69] |  | 0.26 |
|  | G06 | Intracranial and intraspinal abscess and granuloma | NR | <6 | 4.40 [1.67, 11.62] |  | 0.26 |
|  | Z51 | Encounter for other aftercare | 1,269 | 529 | 2.49 [2.25, 2.77] |  | 0.25 |
|  | I67 | Other cerebrovascular diseases | 79 | 17 | 4.65 [2.75, 7.85] |  | 0.23 |
|  | Z54 | Convalescence | 603 | 117 | 5.23 [4.28, 6.38] |  | 0.22 |
| **20** | **Neurology/Pharmacology Emergencies: Epilepsy, seizures, and prescription drugs' poisoning** | | **1,284** | **288** | **4.56 [4.00, 5.19]** | **<0.0001** | **-** |
|  | Y46 | Antiepileptics and antiparkinsonism drugs | NR | <6 | 19.33 [6.06, 61.70] |  | 0.58 |
|  | G40 | Epilepsy and recurrent seizures | 369 | 76 | 4.91 [3.83, 6.29] |  | 0.49 |
|  | T42 | Poisoning by, adverse effect of and underdosing of antiepileptic, sedative- hypnotic and antiparkinsonism drugs | 59 | 25 | 2.36 [1.48, 3.77] |  | 0.45 |
|  | R56 | Convulsions, not elsewhere classified | 864 | 185 | 4.75 [4.05, 5.58] |  | 0.42 |
|  | G41 | Status epilepticus | 55 | 8 | 6.88 [3.27, 14.43] |  | 0.39 |
|  | R27 | Other lack of coordination | 51 | 13 | 3.92 [2.13, 7.21] |  | 0.21 |
| **21** | **Trauma: Assault by sharp object to upper body regions** | | **178** | **33** | **5.39 [3.72, 7.82]** | **<0.0001** | **-** |
|  | X99 | Assault by sharp object | 92 | 16 | 5.75 [3.38, 9.78] |  | 0.74 |
|  | S21 | Open wound of thorax | 30 | 9 | 3.33 [1.58, 7.02] |  | 0.68 |
|  | S11 | Open wound of neck | 39 | 11 | 3.55 [1.82, 6.92] |  | 0.4 |
|  | S15 | Injury of blood vessels at neck level | NR | <6 | NA [NA, NA] |  | 0.22 |
| **22** | **Trauma: Motorcycle injuries** | | **2,133** | **674** | **3.27 [3.00, 3.58]** | **<0.0001** | **-** |
|  | V28 | Motorcycle rider injured in noncollision transport accident | 252 | 58 | 4.34 [3.27, 5.78] |  | 0.63 |
|  | V29 | Motorcycle rider injured in other and unspecified transport accidents | 90 | 14 | 6.43 [3.66, 11.29] |  | 0.59 |
|  | V27 | Motorcycle rider injured in collision with fixed or stationary object | 51 | 6 | 8.50 [3.65, 19.81] |  | 0.47 |
|  | V23 | Motorcycle rider injured in collision with car, pick-up truck or van | 142 | 16 | 8.88 [5.29, 14.88] |  | 0.31 |
|  | S42 | Fracture of shoulder and upper arm | 1,074 | 485 | 2.23 [2.00, 2.49] |  | 0.24 |
|  | V86 | Occupant of special all-terrain or other off-road motor vehicle, injured in transport accident | 742 | 121 | 6.45 [5.29, 7.85] |  | 0.21 |
|  | V22 | Motorcycle rider injured in collision with two- or three-wheeled motor vehicle | NR | <6 | 20.00 [2.68, 149.03] |  | 0.2 |
| **23** | **Neurology/Psychiatry: Alcohol- and drug-related disorders** | | **1,960** | **435** | **4.66 [4.19, 5.18]** | **<0.0001** | **-** |
|  | Y90 | Evidence of alcohol involvement determined by blood alcohol level | NR | <6 | 26.50 [9.77, 71.91] |  | 0.78 |
|  | R78 | Findings of drugs and other substances, not normally found in blood | 115 | 33 | 3.48 [2.37, 5.13] |  | 0.74 |
|  | F10 | Alcohol related disorders | 1,819 | 402 | 4.67 [4.18, 5.21] |  | 0.23 |
| **24** | **Emergency Medicine: Brain and cardiopulmonary emergencies** | | **629** | **76** | **8.28 [6.52, 10.50]** | **<0.0001** | **-** |
|  | G93 | Other disorders of brain | 413 | 20 | 20.65 [13.18, 32.35] |  | 0.53 |
|  | Z52 | Donors of organs and tissues | 24 | 7 | 3.43 [1.48, 7.96] |  | 0.51 |
|  | D65 | Disseminated intravascular coagulation [defibrination syndrome] | NR | <6 | 7.00 [1.59, 30.80] |  | 0.5 |
|  | E23 | Hypofunction and other disorders of the pituitary gland | NR | <6 | 7.80 [3.07, 19.79] |  | 0.38 |
|  | I46 | Cardiac arrest | 213 | 45 | 4.73 [3.43, 6.53] |  | 0.26 |
| **25** | **Trauma: Fractures of lower limb and multiple regions, and intracranial hemorrhage** | | **1,925** | **363** | **5.75 [5.11, 6.46]** | **<0.0001** | **-** |
|  | Z54 | Convalescence | 603 | 117 | 5.23 [4.28, 6.38] |  | 0.43 |
|  | Z50 | Care involving use of rehabilitation procedures | 410 | 66 | 6.38 [4.90, 8.30] |  | 0.33 |
|  | I62 | Other and unspecified nontraumatic intracranial hemorrhage | 772 | 12 | 64.33 [36.38, 113.78] |  | 0.3 |
|  | S72 | Fracture of femur | 421 | 192 | 2.22 [1.87, 2.64] |  | 0.25 |
| **26** | **Trauma: Assault, injuries to orbits, and alcohol-related disorders** | | **6,151** | **762** | **9.30 [8.58, 10.09]** | **<0.0001** | **-** |
|  | Y04 | Assault by bodily force | 3,796 | 93 | 47.29 [37.89, 59.01] |  | 0.59 |
|  | Y09 | Assault by unspecified means | 375 | 6 | 93.25 [34.81, 249.77] |  | 0.47 |
|  | Y00 | Assault by blunt object | NR | <6 | 145.67 [46.80, 453.41] |  | 0.37 |
|  | H05 | Disorders of orbit | 80 | 37 | 2.16 [1.46, 3.19] |  | 0.26 |
|  | H11 | Other disorders of conjunctiva | 108 | 78 | 1.38 [1.03, 1.85] |  | 0.25 |
|  | H53 | Visual disturbances | 245 | 138 | 1.78 [1.44, 2.19] |  | 0.23 |
|  | F10 | Alcohol related disorders | 1,819 | 402 | 4.67 [4.18, 5.21] |  | 0.21 |
|  | Y08 | Assault by other specified means | 96 | 10 | 9.60 [5.01, 18.41] |  | 0.21 |
| **27** | **Hematology: Bone marrow anemias, and disorders of blood coagulation** | | **659** | **455** | **1.46 [1.29, 1.65]** | **<0.0001** | **-** |
|  | D46 | Myelodysplastic syndromes | NR | <6 | 9.75 [3.48, 27.28] |  | 0.63 |
|  | D61 | Other aplastic anemias and other bone marrow failure syndromes | 54 | 18 | 3.00 [1.76, 5.11] |  | 0.59 |
|  | D69 | Purpura and other hemorrhagic conditions | 144 | 85 | 1.69 [1.30, 2.22] |  | 0.54 |
|  | D64 | Other anemias | 482 | 360 | 1.35 [1.17, 1.55] |  | 0.2 |
| **28** | **Psychiatry/Community Health: Functional and economic inquiry, HIV infection, and substance abuse** | | **2,417** | **694** | **3.64 [3.34, 3.97]** | **<0.0001** | **-** |
|  | F14 | Cocaine related disorders | 78 | 19 | 4.11 [2.49, 6.78] |  | 0.48 |
|  | F11 | Opioid related disorders | 73 | 31 | 2.35 [1.55, 3.58] |  | 0.47 |
|  | Z59 | Problems related to housing and economic circumstances | 42 | 19 | 2.21 [1.29, 3.80] |  | 0.4 |
|  | F10 | Alcohol related disorders | 1,819 | 402 | 4.67 [4.18, 5.21] |  | 0.32 |
|  | Z91 | Personal risk factors, not elsewhere classified | 142 | 72 | 1.97 [1.49, 2.62] |  | 0.32 |
|  | Z72 | Problems related to lifestyle | 431 | 157 | 2.79 [2.32, 3.36] |  | 0.26 |
|  | B18 | Chronic viral hepatitis | 53 | 20 | 2.65 [1.58, 4.43] |  | 0.25 |
|  | Z21 | Asymptomatic human immunodeficiency virus [HIV] infection status | 27 | 8 | 3.38 [1.53, 7.43] |  | 0.25 |
| **29** | **Trauma: Superficial injuries** | | **1,813** | **894** | **2.06 [1.90, 2.23]** | **<0.0001** | **-** |
|  | S30 | Superficial injury of abdomen, lower back, pelvis and external genitals | 363 | 149 | 2.45 [2.02, 2.96] |  | 0.47 |
|  | S40 | Superficial injury of shoulder and upper arm | 390 | 218 | 1.79 [1.52, 2.12] |  | 0.41 |
|  | S70 | Superficial injury of hip and thigh | 206 | 111 | 1.86 [1.48, 2.35] |  | 0.38 |
|  | S20 | Superficial injury of thorax | 479 | 219 | 2.20 [1.88, 2.59] |  | 0.32 |
|  | S10 | Superficial injury of neck | 350 | 82 | 4.27 [3.36, 5.43] |  | 0.32 |
|  | S39 | Other and unspecified injuries of abdomen, lower back, pelvis and external genitals | 235 | 153 | 1.54 [1.26, 1.89] |  | 0.23 |
| **30** | **Trauma: Pedal cycle injuries** | | **2,292** | **514** | **4.64 [4.21, 5.12]** | **<0.0001** | **-** |
|  | V18 | Pedal cycle rider injured in noncollision transport accident | 1,258 | 344 | 3.74 [3.32, 4.23] |  | 0.58 |
|  | V19 | Pedal cycle rider injured in other and unspecified transport accidents | 199 | 53 | 3.75 [2.77, 5.08] |  | 0.53 |
|  | T00 | Superficial injuries involving multiple body regions | 586 | 99 | 5.97 [4.82, 7.39] |  | 0.36 |
|  | V13 | Pedal cycle rider injured in collision with car, pick-up truck or van | 340 | 28 | 12.14 [8.26, 17.85] |  | 0.35 |
|  | V17 | Pedal cycle rider injured in collision with fixed or stationary object | NR | <6 | 19.20 [7.81, 47.18] |  | 0.22 |
| **31** | **Trauma: Heavy machinery motor-vehicle accidents** | | **2,661** | **298** | **9.53 [8.42, 10.79]** | **<0.0001** | **-** |
|  | V44 | Car occupant injured in collision with heavy transport vehicle or bus | 145 | 7 | 20.71 [9.70, 44.23] |  | 0.47 |
|  | V54 | Occupant of pick-up truck or van injured in collision with heavy transport vehicle or bus | NR | <6 | 26.00 [3.53, 191.60] |  | 0.43 |
|  | V43 | Car occupant injured in collision with car, pick-up truck or van | 2,298 | 255 | 9.48 [8.30, 10.82] |  | 0.26 |
|  | S19 | Other specified and unspecified injuries of neck | 297 | 50 | 6.15 [4.53, 8.34] |  | 0.23 |
| **32** | **Trauma: Pedestrian injuries and fractures, induced by transport machinery** | | **982** | **92** | **10.89 [8.77, 13.51]** | **<0.0001** | **-** |
|  | V03 | Pedestrian injured in collision with car, pick-up truck or van | 812 | 61 | 13.73 [10.54, 17.88] |  | 0.46 |
|  | V09 | Pedestrian injured in other and unspecified transport accidents | 64 | 8 | 8.00 [3.84, 16.68] |  | 0.43 |
|  | S15 | Injury of blood vessels at neck level | NR | <6 | NA [NA, NA] |  | 0.32 |
|  | T08 | Fracture of spine, level unspecified | NR | <6 | 2.20 [0.76, 6.33] |  | 0.27 |
|  | T02 | Fractures involving multiple body regions | NR | <6 | 21.33 [6.70, 67.90] |  | 0.25 |
|  | I72 | Other aneurysm | 42 | 17 | 2.47 [1.41, 4.34] |  | 0.2 |
| **33** | **Trauma: Falls from elevation** | | **2,147** | **296** | **7.59 [6.70, 8.59]** | **<0.0001** | **-** |
|  | W17 | Other fall from one level to another | 1,235 | 228 | 5.58 [4.83, 6.44] |  | 0.58 |
|  | W13 | Fall from, out of or through building or structure | 311 | 18 | 17.28 [10.74, 27.79] |  | 0.51 |
|  | W11 | Fall on and from ladder | 679 | 53 | 13.27 [9.99, 17.64] |  | 0.35 |
|  | W12 | Fall on and from scaffolding | NR | <6 | 49.00 [6.77, 354.86] |  | 0.22 |
| **34** | **Neurology/Trauma: Sensory medical and functional inquiry, and strike against an object** | | **11,604** | **2,079** | **6.80 [6.45, 7.16]** | **<0.0001** | **-** |
|  | R51 | Headache | 1,464 | 573 | 2.61 [2.36, 2.87] |  | 0.51 |
|  | F07 | Personality and behavioral disorders due to known physiological condition | NR | <6 | NA [NA, NA] |  | 0.47 |
|  | G44 | Other headache syndromes | 124 | 50 | 2.48 [1.79, 3.44] |  | 0.32 |
|  | W22 | Striking against or struck by other objects | 8,386 | 1,203 | 8.13 [7.62, 8.68] |  | 0.32 |
|  | Z02 | Encounter for administrative examination | 236 | 65 | 3.67 [2.79, 4.84] |  | 0.21 |
|  | W20 | Struck by thrown, projected or falling object | 1,480 | 206 | 7.43 [6.41, 8.62] |  | 0.2 |

*Refers to who is the perpetrator of the injury (Factor 15)

# **Supplementary Table 3**: Frequencies, ORs, and factor loadings of codes that met the factor analysis cut-off and rationale for Host, Agent, and Environment designations.

| **Factor number** | **Description** | **Category** | **ICD-10-CA Codes** | **Frequency in cohorts** | | **OR [95% CI]** |
| --- | --- | --- | --- | --- | --- | --- |
|  |  |  |  | **TBI** | **Reference** |  |
| Factor 1 | Multitrauma^H/A^ | Traumatology/Human–product interaction | S36, S27, S37, T06, T79, S42, S26, S72 | 2,535 | 749 | 3.49 [3.21-3.79] |
| Factor 2 | Heart & Metabolic Disorders^H^ | Cardiology | E78, I10, I48, Z95, I50, Z92, E03, E11 | 6,095 | 3,787 | 1.97 [1.88-2.07] |
| Factor 3 | Alzheimer’s & Dementia^H^ | Neurology | F00, G30 | 267 | 79 | 3.41 [2.65-4.39] |
| Factor 4 | Endocrine, Metabolic & Elderly Emerg^H^ | Emergency medicine/ Geriatrics | I10, E87, E83, E22, F05, Z75, N17, F06, B96, I95, R41 | 6,338 | 2,873 | 2.85 [2.70-3.00] |
| Factor 5 | Complications & Resp Emerg^E^ | Emergency medicine | J95, Y84, J15 | 638 | 204 | 3.14 [2.68-3.68] |
| Factor 6 | Elderly Disorders, Neoplasms & Falls^H/A^ | Geriatrics, External Cause of Injury | S72, F05, Z75, R41, F03, Z51, R29, W05, R26, W19, C79, Z74, W06 | 10,793 | 2,275 | 6.92 [6.54-7.31] |
| Factor 7 | Stroke & Brain Emerg Sequelae^H,^ | Neurology | G81, R47, I63, I69, R13 | 929 | 322 | 2.96 [2.61-3.37] |
| Factor 8 | Renal Dysfunction^H^ | Nephrology | N17, Z99, N18, N08 | 845 | 439 | 2.00 [1.77-2.25] |
| Factor 9 | Resp Emerg, Septicemia^E^ | Emergency medicine | N17, J17, A41, J96 | 844 | 378 | 2.32 [2.05-2.63] |
| Factor 10 | Coagulopathies^H^ | Hematology | Z92, Y44, D68 | 826 | 183 | 4.65 [3.95-5.48] |
| Factor 11 | Liver Disorders^H^ | Gastroenterology | K70, R18, K72, B18 | 198 | 71 | 2.79 [2.13-3.66] |
| Factor 12 | Resp Infections & ABX Resistance^E^ | Infectious diseases/ Emergency medical services systems | B96, B95, U82, A49, L89 | 1,147 | 570 | 2.09 [1.88-2.31] |
| Factor 13 | Airway Obstruction^E^ | Emergency medicine/ Emergency medical services systems | Z51, R13, J96, L89, J69, W80 | 1,956 | 771 | 2.70 [2.48-2.95] |
| Factor 14 | Parkinson’s & Dementia^H^ | Neurology | F02, G20, G31 | 221 | 50 | 4.42 [3.25-6.01] |
| Factor 15 | Abuse & Sexual Assault^E^ | Traumatology/Family Medicine | T74, Y07, Y05, Y06 | 196 | 15 | 13.07 [7.73-22.09] |
| Factor 16 | Falls & Syncope^H/A^ | Traumatology/External Cause of Injury | I95, W19, R55, W18, W01 | 15,516 | 3,106 | 6.79 [6.49-7.11] |
| Factor 17 | Diabetic Emergencies^H^ | Emergency medicine/ Emergency medical services systems | E11, N08, E14, R73, G63 | 3,479 | 2,426 | 1.54 [1.45-1.63] |
| Factor 18 | Car Collision^A^ | Injury-producing agent | V43, V49, V48, V89, V47, T14, Z04, V58 | 4,723 | 711 | 7.14 [6.58-7.75] |
| Factor 19 | Brain & Other Hemorrhages^H^ | Neurology | Z51, C79, I61, I60, G91, I62, G06, I67, Z54 | 2,887 | 744 | 4.24 [3.90-4.62] |
| Factor 20 | Seizures & Drug Adversities^H/E^ | Neurology/ Pharmacology emergencies | Y46, G40, T42, R56, G41, R27 | 1,284 | 288 | 4.56 [4.00-5.19] |
| Factor 21 | Assault^A^ | Injury-producing agent | X99, S21, S11, S15 | 178 | 33 | 5.39 [3.72-7.82] |
| Factor 22 | Motorcycle Collision^A^ | Injury-producing agent | S42, V28, V29, V27, V23, V86, V22 | 2,133 | 674 | 3.27 [3.00-3.58] |
| Factor 23 | Alcohol & Drugs Misuse^H^ | Psychiatry | Y90, R78, F10 | 1,960 | 435 | 4.66 [4.19-5.18] |
| Factor 24 | Multiple Systems Collapse^H^ | Neurosurgery | G93, Z52, D65, E23, I46 | 629 | 76 | 8.28 [6.53-10.51] |
| Factor 25 | Intracranial Pathology, Convalescence^H/E^ | Physical medicine and Rehabilitation | S72, I62, Z54, Z50 | 1,925 | 363 | 5.75 [5.11-6.46] |
| Factor 26 | Assault & Alcohol Disorders^H/A^ | Psychiatry/Injury-producing agent | F10, Y04, Y09, Y00, H05, H11, H53, Y08 | 6,151 | 762 | 9.30 [8.58-10.09] |
| Factor 27 | Aplastic Anemias & Hemorrhages^H^ | Hematology/Neurology | D46, D61, D69, D64 | 659 | 455 | 1.46 [1.29-1.65] |
| Factor 28 | Risky Behaviors, Drug Disorders & Social Disparities ^H/E^ | Family medicine/ Psychiatry/Population and community health | B18, F10, F14, F11, Z59, Z91, Z72, Z21 | 2,417 | 694 | 3.64 [3.34-3.97] |
| Factor 29 | Superficial Injuries^H/A^ | Traumatology/Human–product interaction | S30, S40, S70, S20, S10, S39 | 1,813 | 894 | 2.06 [1.90-2.23] |
| Factor 30 | Pedal cycle Injuries^A^ | Injury-producing agent | V18, V19, T00, V13, V17 | 2,292 | 514 | 4.64 [4.21-5.13] |
| Factor 31 | Heavy Transport Injuries^A^ | Injury-producing agent | V43, V44, V54, S19, W10 | 2,661 | 298 | 9.53 [8.42-10.79] |
| Factor 32 | Pedestrian Injuries, Car Collision^A^ | Injury-producing agent | S15, V03, V09, T08, T02, I72 | 982 | 92 | 10.89 [8.78-13.52] |
| Factor 33 | Falls from Elevation^A^ | Injury-producing agent | W17, W13, W11, W12 | 2,147 | 296 | 7.59 [6.70-8.59] |
| Factor 34 | Headache, Blurred Vision & Object Strikes^H/A^ | Psychiatry/Neurology/Injury-producing agent | R51, F07, G44, W22, Z02, W20 | 11,604 | 2,079 | 6.80 [6.45-7.16] |

**Color coding:**

| **Designation** | **Colour** | **Factors** |
| --- | --- | --- |
| Host |  | 2; 3; 4; 7; 8; 10; 11; 14; 17; 19; 23; 24; 27 |
| Agent |  | 18; 21; 22; 30; 31; 32; 33 |
| Environment |  | 5; 9; 12; 13; 15 |
| Host-Agent linked |  | 1; 6; 16; 26; 29; 34 |
| Host-Environment  linked |  | 20; 25; 28 |

**Definition of Host**: The Host factor includes all the variables characterizing the human subjects under study on either the individual or the population level. Variables that may influence one’s susceptibility to injury can be categorized into three groups (1) biological, such as age, sex, race, and genotype; (2) psychosocial, such as personality, education, occupation, marital status, and place of residence; and (3) behavioral, such as risk-taking, alcohol and drug use, seatbelt use, and safety helmet use.

**Definition of Agent**: The Agent describes how the injury-producing agent reaches the host. These are most often products in the environment, and it is the human–product interaction that allows the transfer of energy in injurious amounts. This concept is well illustrated by motor vehicle crashes and falls from ladders, both of which are significant sources of injury.

**Definition of Environment**: The concept of Environment in injury epidemiology is expansive, comprising all the elements constituting the context, circumstance, and conditions that may influence, directly or indirectly, the occurrence of injury. The environmental factor can be generally categorized into three groups: physical, biological, and socioeconomic (Gordon 1949). The physical environment includes both natural features, such as climate, weather, and terrain, and man-made structures and conditions, such as windows, stairs, stoves, lighting, air conditioning, and ventilation. Overall, the physical environment is the most important determinant of injury because it can act as the vector of the etiologic agent, influence the form, duration, and intensity of the energy transferred to the host, and substantially modify the host’s susceptibility to injury. The biological environment, such as population density and fauna, may also play a significant role in the causation of traffic injury and other types of injury (Björnstig 1992; Goldstein et al. 2011). The physical environment is especially relevant to injury prevention and control because it can be most easily changed. The socioeconomic environment is composed of a variety of constructs, such as socioeconomic status, income equality, policy, regulation, law, international treaties, culture, and emergency medical services systems. The socioeconomic environment has become increasingly complex and its relation to injury remains an important topic of epidemiologic research.

**References**

1. GORDON J. E. (1949). The epidemiology of accidents. American journal of public health and the nation's health, 39(4), 504–515.
2. Björnstig U. (1992). Accidents in the north. Some aspects on snowmobile accidents and moose-car collisions. *Arctic medical research*, 51 Suppl 7, 56–58.
3. Goldstein, G. P., Clark, D. E., Travis, L. L., & Haskins, A. E. (2011). Explaining regional disparities in traffic mortality by decomposing conditional probabilities. *Injury prevention : journal of the International Society for Child and Adolescent Injury Prevention*, **17**(2), 84–90.

**Supplementary Figure 1**: Heatmap of correlation between factors during the injury event-phase period for patients with TBI in the training dataset. Factors were clustered using a hierarchical clustering with Ward (minimum variance) linkage. For the left heatmap, the lower half of the correlation matrix displays male-specific correlations, and the upper half displays female-specific correlations. Red squares indicate a positive correlation, and blue squares indicate a negative correlation between two factors. The right heatmap displays the difference in correlations between males and females. Thus, the interpretation differs in that red squares indicate that females have a more positive correlation (closer to 1) than males, and blue squares indicate that males have a more positive correlation than females. In both heatmaps, white squares indicate correlations that were insignificant after hypothesis testing with multiple testing correction using the Benjamini-Yekutieli method.


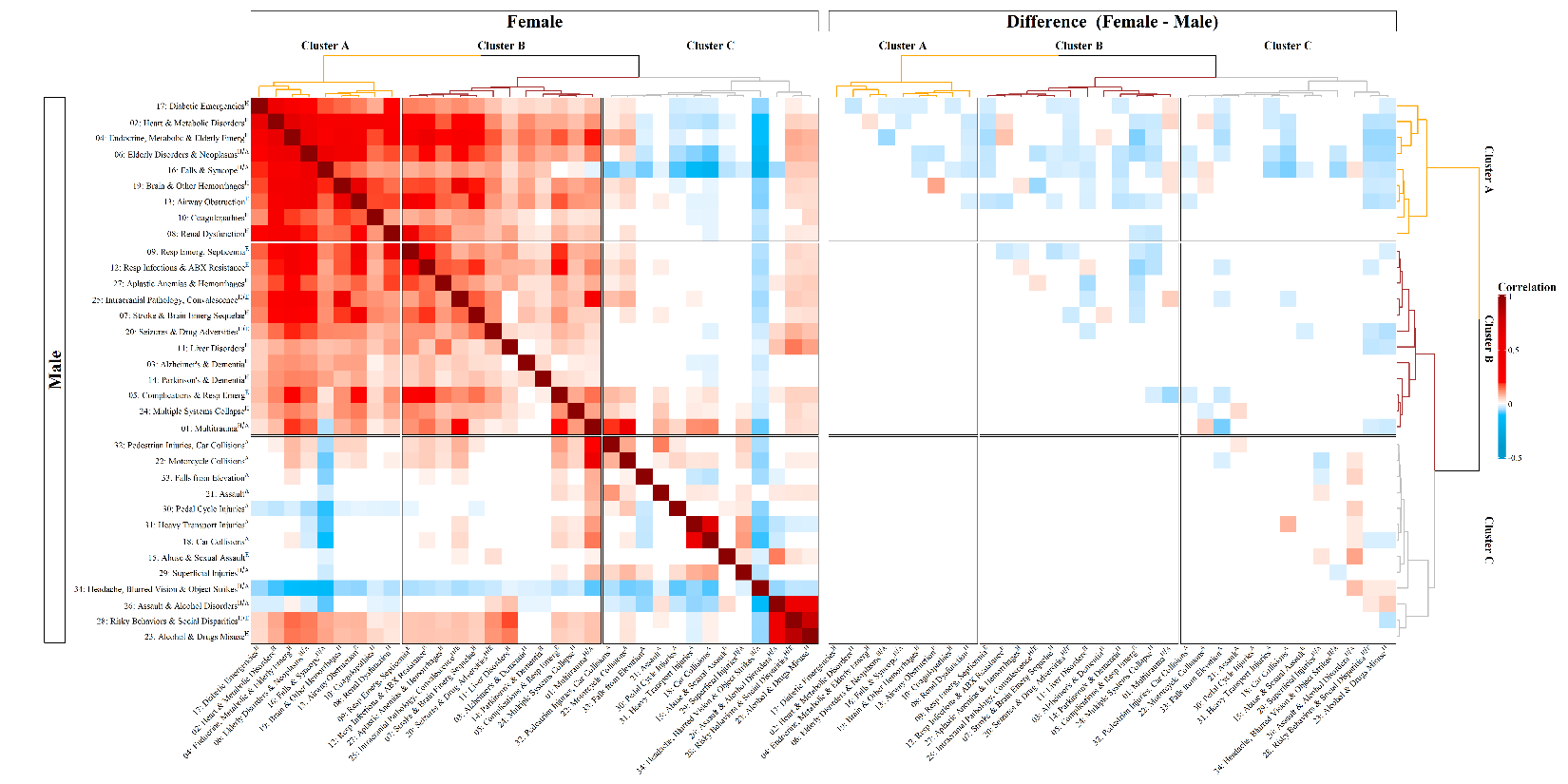


**Supplementary Figure 2**: Heatmap of correlation between factors during the injury event-phase period for patients with TBI in the validation dataset. Factors were clustered using a hierarchical clustering with Ward (minimum variance) linkage. For the left heatmap, the lower half of the correlation matrix displays male-specific correlations, and the upper half displays female-specific correlations. Red squares indicate a positive correlation, and blue squares indicate a negative correlation between two factors. The right heatmap displays the difference in correlations between males and females. Thus, the interpretation differs in that red squares indicate that females have a more positive correlation (closer to 1) than males, and blue squares indicate that males have a more positive correlation than females. In both heatmaps, white squares indicate correlations that were insignificant after hypothesis testing with multiple testing correction using the Benjamini-Yekutieli method.


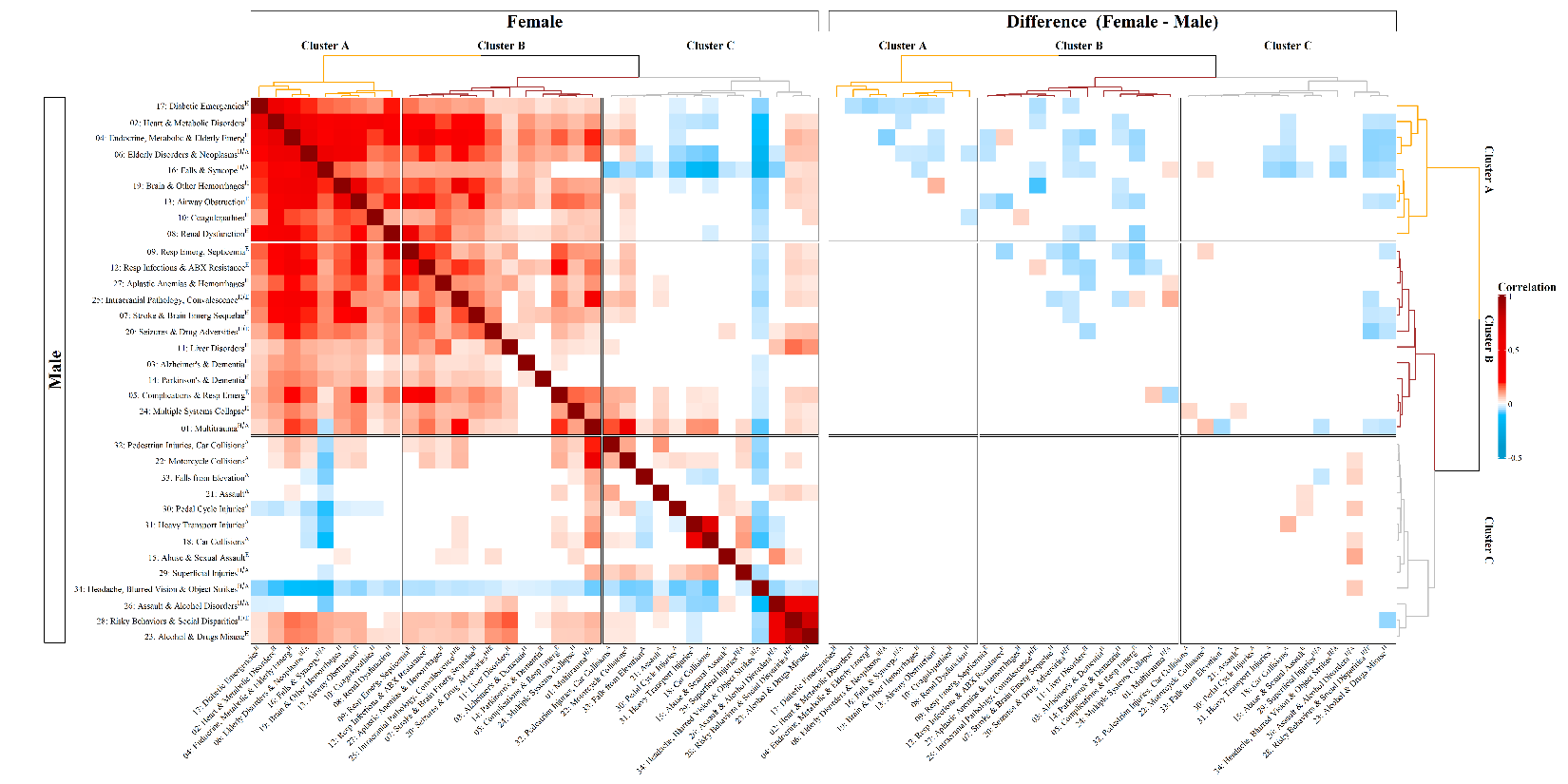


**Supplementary Figure 3**: Heatmap of correlation between Haddon Matrix-classified factors and external cause as well as TBI severity for patients with TBI in the training set. The leftmost, middle, and rightmost heatmaps display the correlations for females, males, and their differences, respectively. For the sex-specific heatmaps, red squares indicate a positive correlation, and blue squares indicate a negative correlation between two factors. For the differences heatmap, red squares indicate that females have a more positive correlation (closer to 1) than males, and blue squares indicate that males have a more positive correlation than females. White squares indicate correlations that were insignificant after hypothesis testing with multiple testing correction using the Benjamini-Yekutieli method.


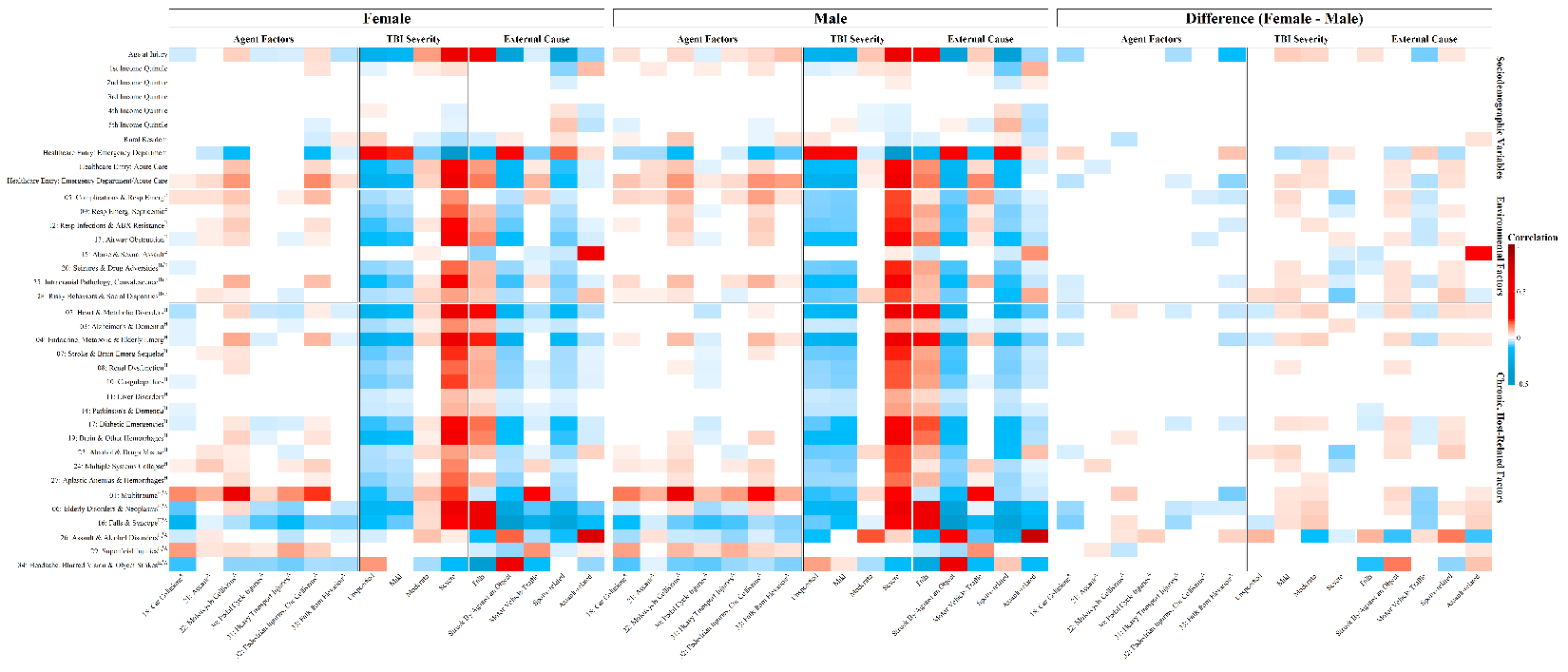


**Supplementary Figure 4**: Heatmap of correlation between Haddon Matrix-classified factors and external cause as well as TBI severity for patients with TBI in the validation set. The leftmost, middle, and rightmost heatmaps display the correlations for females, males, and their differences, respectively. For the sex-specific heatmaps, red squares indicate a positive correlation, and blue squares indicate a negative correlation between two factors. For the differences heatmap, red squares indicate that females have a more positive correlation (closer to 1) than males, and blue squares indicate that males have a more positive correlation than females. White squares indicate correlations that were insignificant after hypothesis testing with multiple testing correction using the Benjamini-Yekutieli method.


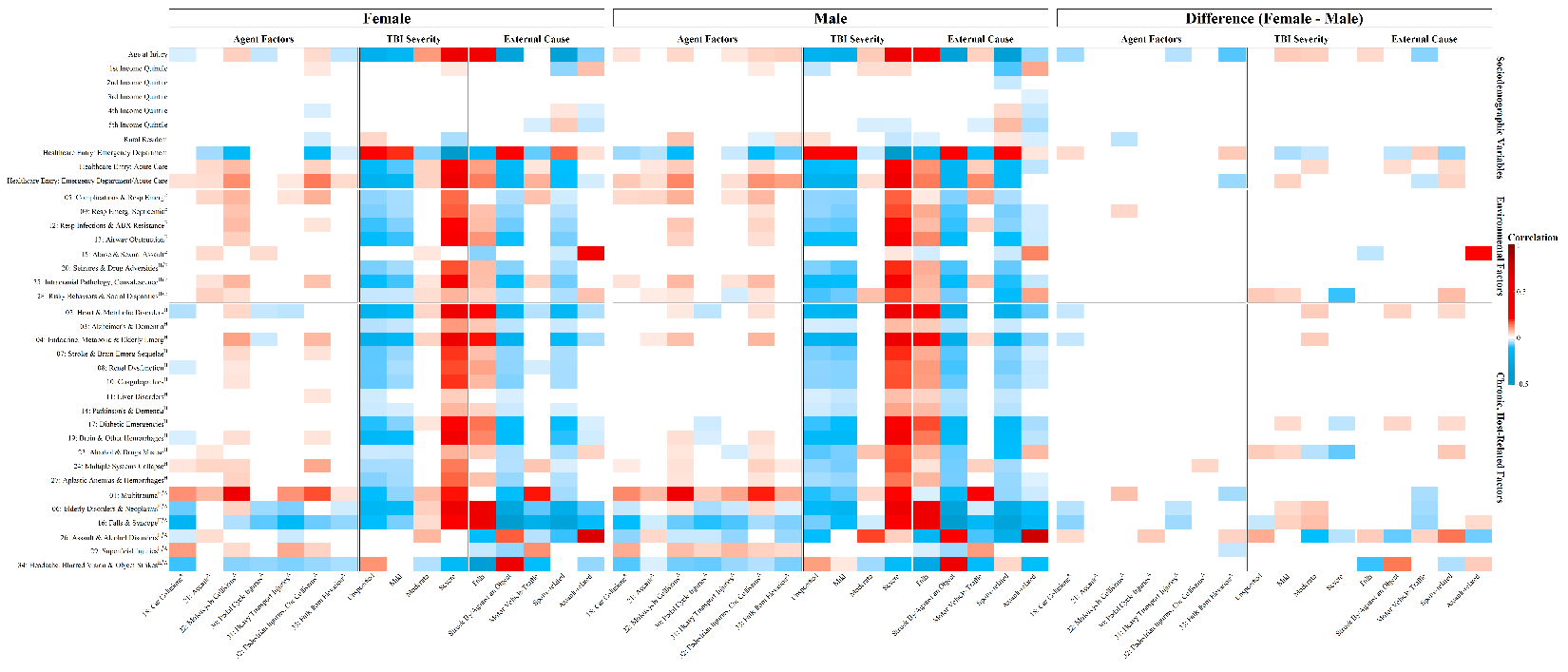

Supplement: Supplementary file 1 — Additional file 1: Supplementary Table 1. Data-driven studies examining TBI event, with results presented by the Haddon Matrix designations. Supplementary Table 2. Frequencies, ORs, and factor loadings of codes that met the factor analysis cut-off. Supplementary Table 3. Frequencies, ORs, and factor loadings of codes that met the factor analysis cut-off and rationale for Host, Agent, and environment designations. Supplementary Figure 1. Heatmap of correlation between factors during the injury event-phase period for patients with TBI in the training dataset. Supplementary Figure 2. Heatmap of correlation between factors during the injury event-phase period for patients with TBI in the validation dataset. Supplementary Figure 3. Heatmap of correlation between Haddon Matrix-classified factors and external cause as well as TBI severity for patients with TBI in the training set. Supplementary Figure 4. Heatmap of correlation between Haddon Matrix-classified factors and external cause as well as TBI severity for patients with TBI in the validation set. [file 12874_2021_1493_MOESM1_ESM.docx]
